# Global transcriptional profiles of beating clusters derived from human induced pluripotent stem cells and embryonic stem cells are highly similar

**DOI:** 10.1186/1471-213X-10-98

**Published:** 2010-09-15

**Authors:** Manoj K Gupta, Damir J Illich, Andrea Gaarz, Matthias Matzkies, Filomain Nguemo, Kurt Pfannkuche, Huamin Liang, Sabine Classen, Michael Reppel, Joachim L Schultze, Jürgen Hescheler, Tomo Šarić

**Affiliations:** 1 Center for Physiology and Pathophysiology, Institute for Neurophysiology, University of Cologne, Robert-Koch-Str. 39, 50931 Cologne, Germany; 2 LIMES (Life and Medical Sciences Bonn), Program Unit Molecular Immune & Cell Biology, Laboratory for Genomics and Immunoregulation, University of Bonn, Germany; 3 Center for Molecular Medicine Cologne, University of Cologne, Cologne, Germany; 4 Department of Physiology, Huazhong University of Science and Technology, Tongji Medical College, China; 5 Department of Cardiology, Medical University of Lübeck, Lübeck, Germany

## Abstract

**Background:**

Functional and molecular integrity of cardiomyocytes (CMs) derived from induced pluripotent stem (iPS) cells is essential for their use in tissue repair, disease modelling and drug screening. In this study we compared global transcriptomes of beating clusters (BCs) microdissected from differentiating human iPS cells and embryonic stem (ES) cells.

**Results:**

Hierarchical clustering and principal component analysis revealed that iPS-BCs and ES-BCs cluster together, are similarly enriched for cardiospecific genes and differ in expression of only 1.9% of present transcripts. Similarly, sarcomeric organization, electrophysiological properties and calcium handling of iPS-CMs were indistinguishable from those of ES-CMs. Gene ontology analysis revealed that among 204 genes that were upregulated in iPS-BCs vs ES-BCs the processes related to extracellular matrix, cell adhesion and tissue development were overrepresented. Interestingly, 47 of 106 genes that were upregulated in undifferentiated iPS vs ES cells remained enriched in iPS-BCs vs ES-BCs. Most of these genes were found to be highly expressed in fibroblasts used for reprogramming and 34% overlapped with the recently reported iPS cell-enriched genes.

**Conclusions:**

These data suggest that iPS-BCs are transcriptionally highly similar to ES-BCs. However, iPS-BCs appear to share some somatic cell signature with undifferentiated iPS cells. Thus, iPS-BCs may not be perfectly identical to ES-BCs. These minor differences in the expression profiles may occur due to differential cellular composition of iPS-BCs and ES-BCs, due to retention of some genetic profile of somatic cells in differentiated iPS cell-derivatives, or both.

## Background

Reprogramming of adult somatic cells to induced pluripotent stem (iPS) cells by overexpression of a defined set of transcription factors represents a significant breakthrough in stem cell research [1-6]. One important prerequisite for scientific and therapeutic application of iPS cells is that they can efficiently differentiate into specific functionally and molecularly intact cell lineages. Initial studies have demonstrated that diverse types of mature cell derivatives of all three embryonic germ layers can be differentiated from iPS cells [7-12]. Among these differentiated cells, cardiomyocytes (CMs) represent the most intensively studied cell type [13-15]. Detailed electrophysiological analyses of murine [7,16-19] and human [20-26] iPS cell-derived CMs (iPS-CMs) demonstrated that they are functionally intact and have similar properties to CMs derived from conventional ES cells (ES-CMs).

Although comparisons between iPS cells and conventional ES cells revealed that they have highly similar growth characteristics, gene expression profiles, epigenetic status and developmental potential [3,5,6,27,28], recent comprehensive analyses of various undifferentiated ES and iPS cell lines showed that iPS cells may not be perfectly identical to conventional ES cells at the molecular level [29-31]. These studies demonstrated that iPS cells are characterized by a unique gene and miRNA expression signature as well as a CpG methylation pattern, which distinguish them from ES cells. However, the comparison of global transcriptomes of mature cells differentiated from ES and iPS cells has not yet been performed, and it is not clear whether molecular differences between iPS and ES cells are retained upon their differentiation into mature cells.

Full transcriptional profiles of human ES-CMs have been reported by several groups. Synnergren and coworkers differentiated the human ES cell line SA002 to cardiac lineage in an embryoid body (EB) system and CMs were enriched by mechanical dissection of spontaneously beating clusters (BCs) [32]. The results of this study indicated that human ES-BCs, despite being composed of different cell types, are highly enriched for CM-specific transcripts and display high similarities to human fetal heart tissue. Cao and coworkers reported the transcriptional profile of Percoll density gradient-enriched human H9 ES-CMs that were isolated by centrifugation to a purity of about 45% [33], and most recently, microarray analyses were carried out with highly purified CMs (> 99%) that were generated by drug selection from the transgenic human ES cell line HES3 [34] and H9 [35]. These studies provide the first comprehensive characterization of the molecular signature of purified human ES-CMs revealing high similarities between expression profiles of ES-CMs and human native CMs.

The purpose of this study was to determine the degree of molecular similarity between CMs and other differentiated cell types present in microdissected BCs derived from human iPS cells and conventional ES cells. Our data indicates that iPS-BCs are transcriptionally highly similar to ES-BCs. However, iPS-BCs appear to share some somatic cell signature with undifferentiated iPS cells and express higher levels of transcripts encoding for some extracellular matrix components and cell adhesion molecules than ES-BCs. This expression profile may reflect either the differential cellular composition or activity in iPS- and ES-BCs, partial retention of genetic signature of somatic cells in differentiated iPS cell-derivatives, or both. These findings emphasize the necessity for a careful assessment of the differentiation capacity of iPS cells of various somatic cell origins and molecular profiles of specific mature iPS and ES cell-derivatives, so as to determine whether any alterations may exist that could affect their use for regenerative medicine and research.

## Results

### Cardiac differentiation of human iPS and ES cells

The human iPS cell line derived from foreskin fibroblasts, clone 1 (C1), [6] and the human ES cell line HES-2 were differentiated to CMs using the END2 co-culture system [15] (Additional file 1, Figure S1). Upon inducing differentiation, spontaneously contracting clusters were first observed at day 11 in cultures of both cell lines. At day 15 of differentiation, the fraction of clusters that exhibited spontaneous contractions in iPS and ES cell cultures was 12.8 ± 3.5% and 16.0 ± 4.8%, respectively (n = 9, p = 0.128), (Additional file 1, Figure S2A). With further cultivation no additional beating areas appeared. In BCs derived from iPS and ES cells the CMs represented, respectively, 16.7 ± 5.1% and 15.0 ± 2.6% of all cells (Additional file 1, Figure S2B).

The contraction rates of these BCs were similar in iPS and ES cell cultures (46 ± 6.8 beats/min for iPS cells and 43 ± 4.5 beats/min for ES cells), (Additional file 1, Figure S2C). These data indicate that the iPS cell line Foreskin C1 differentiates to CMs at rates comparable to the ES cell line HES-2 and possesses similar beating characteristics.

### Expression of cardiospecific genes and sarcomeric proteins in BCs

**Figure 1:**
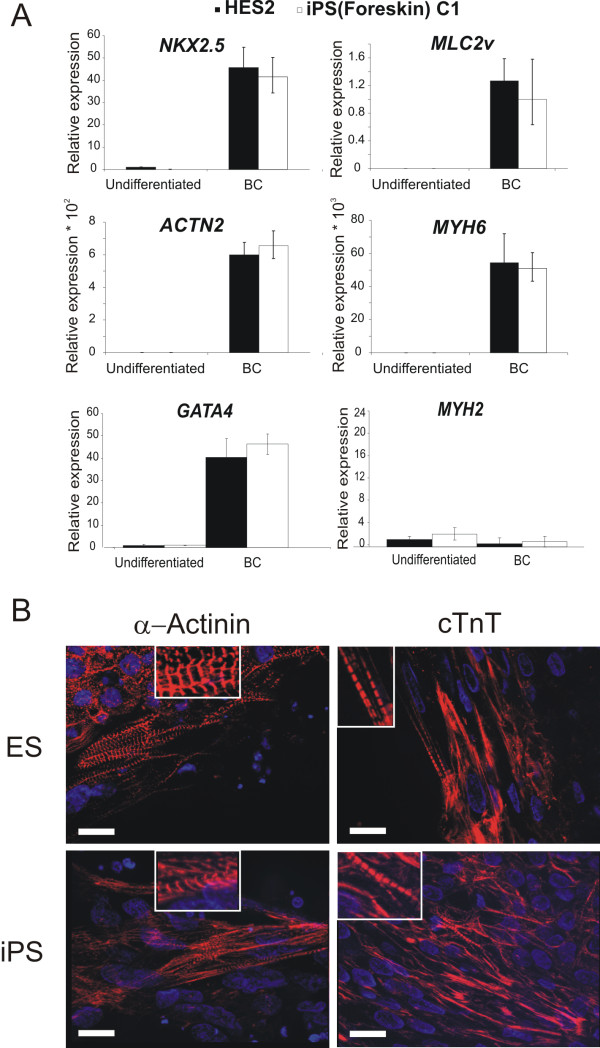
**Expression of cardiac transcripts and proteins in cardiomyocytes derived from human iPS and ES cells**. **A**. qRT-PCR assessment of expression of cardiac specific genes *NKX2.5, MLC2v, ACTN2*, *MYH6, GATA4 *and a skeletal muscle gene *MYH2 *in undifferentiated iPS and ES cells and their corresponding beating clusters (BC) microdissected at day 18 of differentiation. The data are means of triplicate analyses ± SEM. **B**. Anti-sarcomeric cardiac actinin and cardiac troponin T stainings in HES2- (upper panels) and iPS(Forskin) C1-derived (lower panels) cardiomyocytes. Spontaneously beating outgrowths were microdissected at day 15 of differentiation and replated on fibronectin-coated plates. Adherent beating clusters were used for staining 3-5 days later. Scale bar: 20 μm.

To confirm the presence of CMs in BCs isolated from differentiating iPS and ES cells we examined the expression of cardiospecific markers using qRT-PCR (primer sequences are given in Additional file 1, Table S1) and immunocytochemistry. The data on expression of the endogenous and viral pluripotency marker *OCT4 *in undifferentiated and differentiated iPS and ES cells is discussed in Additional file 1 (Figure S3 and Supplementary results). The expression of mRNAs for transcription factors *NKX2.5 *and *GATA4*, and the cardiac structural proteins myosin light chain 2 ventricular isoform (*MLC2v*), α-actinin (*ACTN2*) and α-myosin heavy chain (*MYH6*) was undetectable or very low in undifferentiated cells (Figure 1A). However, their expression was strongly upregulated in BCs at day 18 of differentiation (Figure 1A). The specific marker of skeletal muscle, myosin heavy chain 2 (*MYH2*), was not detectable in these aggregates (Figure 1A). Similar levels of the cardiac genes in microdissected BCs of both cell types indicate that they contain comparable amounts of CMs, which is in agreement with the CM-counting data in the Additional file 1, Figure S2B. Immunocytochemical analyses revealed that CMs in day 18 iPS- and ES-BCs stain positively for cardiac proteins α-actinin and troponin T (cTnT) and display typical pattern of cross-striations indicative of sarcomeric organization in CMs (Figure 1B). The highly ordered striated pattern is highlighted in high resolution images and clearly recapitulates the normal architecture of the contractile apparatus in functional CMs.

### Intact adrenergic and muscarinergic signaling in iPS-CMs

**Figure 2:**
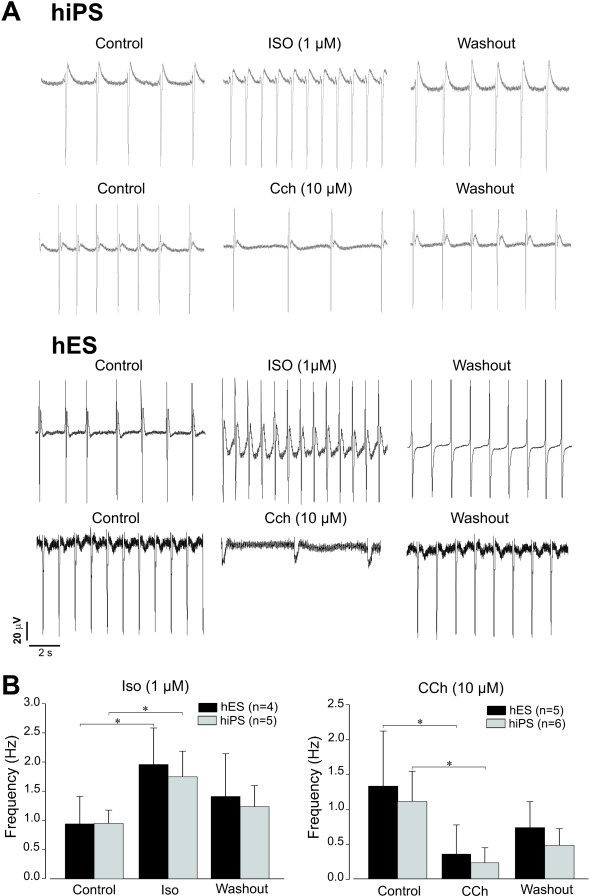
**Electrophysiological analysis by multielectrode arrays (MEAs)**. **A**. Representative field potential (FP) recordings of human iPS- and ES-BCs in the absence or presence of the alpha-adrenergic agonist isoproterenol (ISO, 1 μmol/L) or muscarinic agonist carbachol (CCh, 10 μmol/L). **B**. Statistical analysis of FP frequencies in MEA measurements. Data are shown as means of ± SEM. * p < 0.05.

MEA measurements were performed to verify the functional integrity of CMs in microdissected BCs. In the absence of pharmacological stimulation, iPS-CMs and ES-CMs exhibited similar spontaneous electrical activity with the field potential (FP) frequencies of 1.04 ± 0.39 Hz (n = 11) and 1.15 ± 0.66 Hz (n = 9), respectively (Figure 2 and Additional file 1, Table S2). Application of β-adrenergic receptor agonist isoproterenol (ISO) significantly increased the basal FP frequency in both cell populations to 1.75 ± 0.44 Hz (iPS cells, n = 5) and 1.96 ± 0.63 Hz (ES cells, n = 4), whereas the treatment with the muscarinic receptor agonist carbachol (CCh) led to its reduction in iPS-BCs and ES-BCs to 0.23 ± 0.21 Hz (n = 6) and 0.35 ± 0.42 Hz (n = 5), respectively (Figure 2, and Additional file 1, Table S2). These results suggest that iPS-CMs express functional β-adrenergic and muscarinic receptors and their associated signaling pathways in a manner indistinguishable from ES-CMs.

### Spontaneous Ca^2+ ^transients in iPS-CMs

**Figure 3:**
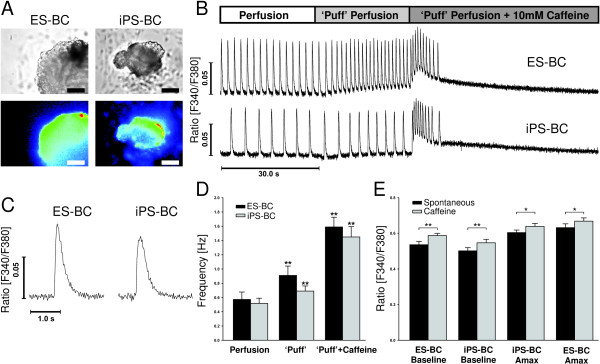
**Spontaneous Ca^2+ ^transients of HES-2 and iPS(Foreskin) C1-derived contracting cell clusters**. **A**. Transmission light images of two beating cell aggregates derived from ES and iPS cells that were loaded with Fura-2 AM (upper row) and the corresponding emitted fluorescence light presented in pseudo color. (Scale bar = 100 μm). **B**. Response of spontaneously beating aggregates derived by both cell lines to caffeine (10 mM) treatment indicates the existence of caffeine-releasable Ca^2+ ^stores in this stage of development (up to 2 weeks after onset of beating). Two representative experiments are shown: 30 s tyrode perfusion followed by 'puff' perfusion of tyrode directly in front of the cells for the same time period with subsequent caffeine application. Both cell types temporally show a caffeine evoked increase of basal, systolic and diastolic [Ca^2+^]_i_. However the Ca^2+ ^amplitude is reduced, accompanied by an increase in beating frequency. **C**. Representative single tracings. The transients show statistically identical characteristics regarding basal Ca^2+ ^level, amplitude, maximum upstroke velocity (V_max, upstroke_) and maximum decay velocity (V_max, decay_) with p > 0.05 (data not shown)**. D**+**E **Caffeine response (n = 7 for iPS-CMs; n = 7 for ES-CMs). Beating frequency is increased by 'puff'-perfusion and subsequent caffeine application in both celltypes (D). Basal calcium levels and the maximum value (A_max_) are raised significantly after caffeine treatment (E). * = p < 0.05; ** = p < 0.01. Additional analyses of these measurements are provided in Additional file 1, Tables S3 and S4.

To further characterize the functional properties of iPS-CMs, we have compared spontaneous Ca^2+ ^release in BCs of human ES and iPS origin using fluorometric Ca^2+ ^imaging with Fura-2 AM (Figure 3A). Both cell types showed rhythmic Ca^2+ ^transients with identical baseline and amplitude, maximum upstroke- and maximum decay velocity, time to peak, total transient time and time to half peak relaxation (Figure 3B, 3C and Additional file 1, Table S3). Simultaneous observation of contractions and Ca^2+ ^transients revealed that each Ca^2+ ^transient was accompanied by a single contraction of the examined iPS- and ES-BCs, being indicative of an intact cardiac excitation-contraction coupling (Additional file 1, Table S4 and Supplementary results). In order to determine whether functional Ca^2+ ^stores in the sarcoplasmic reticulum (SR) are expressed in iPS-CMs we investigated the effects of caffeine on the cells. In all experiments increased beating frequency was observed due to the mechanical stimulation caused by the control Tyrode perfusion with the 'puff'-device (Figure 3B, 3D; p < 0.01). Upon caffeine application an additional short-term increase of the frequency of spontaneous Ca^2+^-release occurred (Figure 3B, 3D). Concomitantly, the magnitude of the transients decreased and the basal Ca^2+ ^level increased (Figure 3B, 3E; p < 0.01). Increased Ca^2+ ^levels in a transient response to caffeine indicate the presence of functional SR Ca^2+^-stores in iPS-CMs.

### Comparative global transcriptome analysis

**Figure 4:**
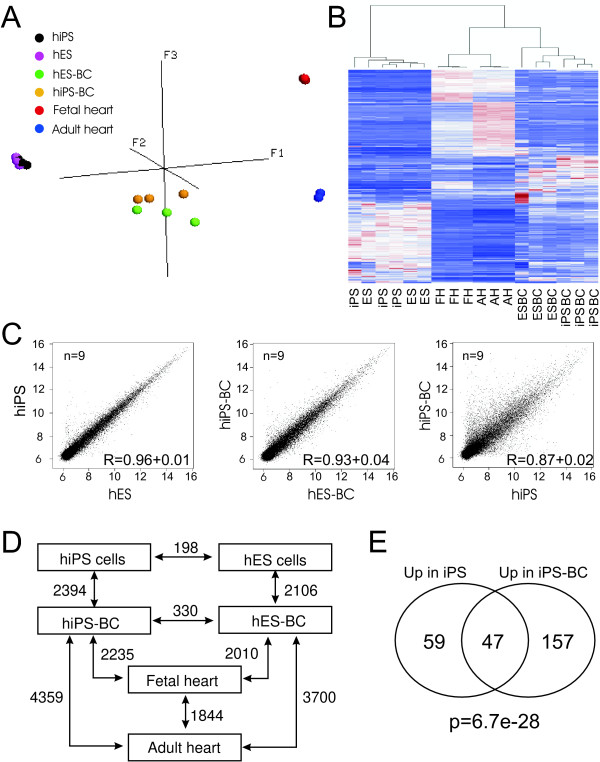
**Global transcriptional analysis of human iPS and ES cells and their corresponding beating clusters**. **A**. Principal component analysis of 4254 variant genes. **B**. Hierarchical clustering analysis of variant genes. **C**. Scatter-plots comparing global gene expression profiles between undifferentiated ES and iPS cells, iPS-BCs and ES-BCs and undifferentiated iPS cells and iPS-BCs. **D**. The number of differentially expressed genes (p < 0.05, 2- fold change) in various intergroup comparisons. **E**. Venn diagram of iPS cell-upregulated (compared to ES cells) and iPS-BC-upregulated (compared to ES-BCs) transcripts and the number of overlapping genes. The p-value has been calculated using the Fisher's exact test.

Functional analyses presented above and those reported by others [20-24,26] indicate that iPS-CMs are electrophysiologically highly similar to those of their ES cell counterparts. To establish whether this functional similarity is mirrored at the molecular level, we compared the global gene expression profiles of undifferentiated ES and iPS cells and beating areas of differentiated iPS and ES cells microdissected at day 18 of differentiation. Fetal (FH) and adult human hearts (AH) were included to serve as positive controls for cardiac transcripts. Samples used for transcriptional analyses are described in Additional file 1, Table S5. All data have been deposited in NCBI's Gene Expression Omnibus and are accessible through GEO Series accession number GSE17579 http://www.ncbi.nlm.nih.gov/geo/query/acc.cgi?token=tzqzjeawkmamedu&acc=GSE 17579. Among expressed transcripts, 4254 probe sets (25.3%) were identified as variable across samples (Additional file 2, Table S6). Principal component analysis (PCA) with this data set showed that samples cluster in four groups corresponding to a) undifferentiated ES and iPS cells, b) microdissected ES- and iPS-BCs, c) fetal heart and d) adult heart (Figure 4A). Hierarchical clustering also showed that undifferentiated iPS cells cluster together with undifferentiated ES cells and iPS-BCs together with ES-BCs (Figure 4B). The transcriptional profiles of FH and AH were more related to those of iPS-BCs and ES-BCs than to those of their undifferentiated counterparts. Furthermore, scatter-plot analysis of every probe set on the array further emphasized a close correlation of gene expression levels between iPS and ES cells (r^2 ^= 0.96 ± 0.01, n = 9) as well as between iPS-BCs and ES-BCs (r^2 ^= 0.93 ± 0.04, n = 9) (Figure 4C). In contrast, correlation between iPS cells and iPS-BCs as well as between undifferentiated ES cells and ES-BCs was significantly lower (r^2 ^= 0.87 ± 0.02, n = 9). A closer look at differentially expressed genes (p-value < 0.05; > 2-fold change; difference between mean intensity signals > 100) revealed that only 198 probe sets (1.19% of present transcripts or 4.65% of variable genes) significantly differ in their expression levels between ES and iPS cells and only 330 probe sets (1.91% of present transcripts and 7.76% of variable genes) were differentially expressed between ES-BCs and iPS-BCs (Figure 4D). Complete lists of up- and down-regulated genes in these comparisons are provided in Additional file 3 (Tables S7 and S8) and Additional file 4 (Tables S9 and S10).

### Genes commonly upregulated in iPS cells and iPS-BCs

In order to determine whether any of the 106 unique transcripts found to be overexpressed in iPS cells in iPS- versus ES-cell comparison also remain overexpressed in iPS-BCs compared to ES-BCs we searched for overlapping genes in these two lists of overexpressed genes and found that among 106 unique iPS cell-enriched transcripts 47 (44.3%) were also present on the list of 204 iPS-BC-enriched transcripts (Figure 4E and Table 1). Using Fisher's exact test we have determined that this overlap was highly statistically significant (p = 6,7e-28). Moreover, 38 (80.9%) of these overlapping transcripts remained expressed at similar levels in undifferentiated iPS cells and differentiated iPS-BCs (Table 1). The gene ontology (GO) analysis of iPS cell-upregulated transcripts did not reveal significant enrichment for any GO-term in this group of genes (data not shown).

**Table 1:** List of 48 genes commonly upregulated in human iPS cells and human iPS-beating clusters (BCs) as compared to, respectively, human ES cells and human ES-BCs.

Symbol^1^	Mean ES	Mean ES-BC	p-value^2 ^(ES vs ES-BC)	Mean iPS	Mean iPS-BC	p-value^2 ^(iPS vs iPS-BC)	Fold change iPS-BC/ES-BC	Overlap^3^
**ADFP**	94	118	> 0,05	2125	1490	> 0,05	12,6	-

**ANGEL2**	510	430	> 0,05	1181	1198	> 0,05	2,8	-

**B3GALNT1**	81	78	> 0,05	386	443	> 0,05	5,7	-

**C1orf176**	95	94	> 0,05	229	231	> 0,05	2,5	-

**CFLAR**	120	92	> 0,05	294	215	> 0,05	2,3	Chin-E

**CKLF**	417	290	> 0,05	921	582	> 0,05	2,0	-

**COMT**	257	194	> 0,05	1172	1958	> 0,05	10,1	Chin-E; Marchetto

**CYorf15A**	123	94	> 0,05	566	407	> 0,05	4,3	-

**CYorf15B**	78	85	> 0,05	237	189	> 0,05	2,2	-

**DNAJC15**	112	179	> 0,05	504	402	> 0,05	2,2	Chin-E

**EIF1AY**	75	77	> 0,05	1569	2114	> 0,05	27,5	-

**EPSTI1**	155	136	> 0,05	345	651	> 0,05	4,8	Chin-E

**HLA-B**	203	158	> 0,05	614	513	> 0,05	3,2	Chin-E

**IRAK1**	97	92	> 0,05	511	480	> 0,05	5,2	-

**JARID1D**	83	79	> 0,05	407	489	> 0,05	6,2	-

**LEMD3**	516	480	> 0,05	1382	1278	> 0,05	2,7	-

**MAP1LC3A**	76	91	> 0,05	753	1276	> 0,05	14,0	Chin-E

**MEIS3P1**	87	112	> 0,05	305	362	> 0,05	3,2	-

**MGC3207**	430	299	> 0,05	1040	757	> 0,05	2,5	-

**MGMT**	83	76	> 0,05	453	405	> 0,05	5,3	-

**MNS1**	89	93	> 0,05	325	217	> 0,05	2,3	-

**NAPRT1**	196	190	> 0,05	679	382	> 0,05	2,0	-

**NLGN4Y**	79	80	> 0,05	385	325	> 0,05	4,1	-

**NME4**	89	127	> 0,05	7001	5461	> 0,05	43,0	Chin-L; Marchetto

**OXCT1**	90	94	> 0,05	272	358	> 0,05	3,8	-

**PTGR1**	84	79	> 0,05	1613	1290	> 0,05	16,3	Chin-E

**PUS7L**	92	82	> 0,05	327	211	> 0,05	2,6	-

**RPS4Y1**	86	96	> 0,05	4190	4645	> 0,05	48,4	-

**SLC39A8**	86	91	> 0,05	263	269	> 0,05	3,0	Marchetto

**SPIN3**	84	96	> 0,05	335	206	> 0,05	2,1	-

**TFCP2**	355	190	> 0,05	765	466	> 0,05	2,5	-

**TMBIM4**	223	232	> 0,05	1848	1545	> 0,05	6,7	Chin-L

**TRIM4**	87	79	> 0,05	307	340	> 0,05	4,3	-

**TSPYL5**	79	82	> 0,05	296	350	> 0,05	4,3	Chin-E, L

**ZNF167**	89	76	> 0,05	262	232	> 0,05	3,1	Marchetto

**ZNF22**	123	256	0,0003	1807	1311	> 0,05	5,1	-

**ZNF248**	95	76	> 0,05	202	212	> 0,05	2,8	Chin-E

**ZNF626**	79	77	> 0,05	196	239	> 0,05	3,1	-

CXCL12	399	958	> 0,05	815	1782	0,0245	1,9	**-**

DYNLT3	82	96	> 0,05	227	455	0	4,7	Chin-E

GGCT	92	179	> 0,05	1908	836	0,0007	4,7	**-**

GRTP1	85	82	> 0,05	571	201	0	2,5	**-**

PHF11	79	79	> 0,05	651	307	0	3,9	**-**

PRKAR1A	83	83	> 0,05	651	1357	0,0003	16,3	**-**

RPL39L	977	155	0	2352	668	0	4,3	**-**

ZNF280D	78	78	> 0,05	511	178	0,0138	2,3	Chin E

KDELR3	79	92	> 0,05	185	488	0,0004	5,3	Chin-E

RPL39L	221	107	> 0,05	997	280	0	2,6	**-**

						Mean ± SD	6,9 ± 9,4	

^1 ^Genes with names depicted in bold are expressed at similar levels in iPS cells and iPS cell-derived beating clusters (iPS-BCs).

^2 ^The p-value of > 0.05 indicates that the differences in gene expression levels in comparisons iPS vs iPS-BC and ES vs ES-BC was not statistically significant. For differentially expressed genes in these comparisons the exact p-value is indicated. All genes in this table were differentially expressed (p < 0.05, > 2-fold change) in comparisons iPS vs ES and iPS-BC vs ES-BC.

^3 ^Overlap with iPS cell-specific genes reported by Chin et al. [29] and/or Marchetto et al. [31] is indicated. Two genes (*NME4 *and *COMT*) in this table were reported to be overexpressed in human iPS cells by both groups. One gene (*TSPYL5*) from our list was reported to be overexpressed in both early and late passage iPS cells by Chin et al. ("Chin-E, L"). The gene *TMBIM4 *was found to be overexpressed only in late passage iPS cells (three different cell lines) by Chin et al. ("Chin-L"). All other probe sets in this table were detected only in early passage iPS cells (five different cell lines) by Chin et al. ("Chin-E").

**Figure 5:**
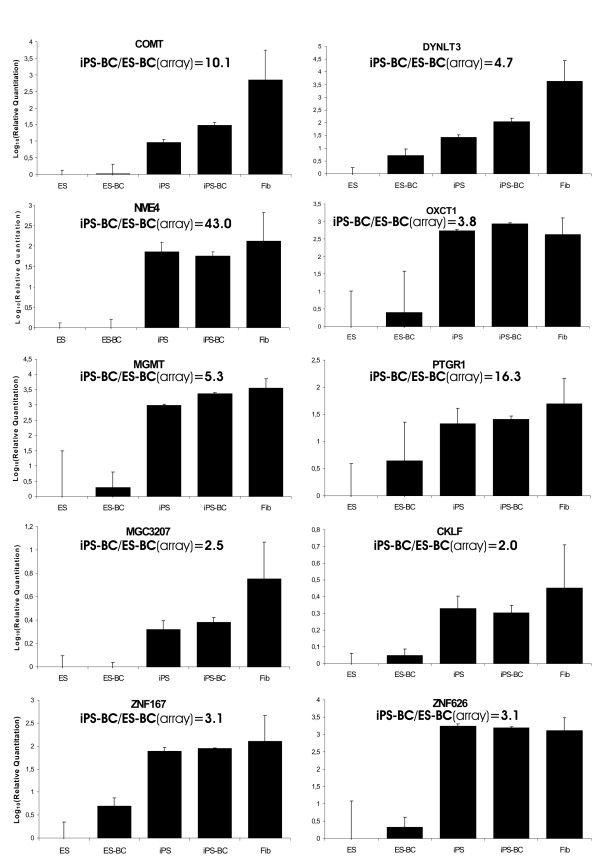
**Quantitative RT-PCR validation of selected genes commonly upregulated in iPS cells and iPS-BCs**. Expression levels of genes indicated above each panel were determined by qRT-PCR in undifferentiated iPS and ES cells, iPS-BCs and ES-BCs as well as in human foreskin fibroblasts. Data for each gene are presented as expression levels relative to undifferentiated human ES cells and are given as the mean ± SEM of triplicate measurements. For comparison, the ratio of signal intensities between iPS-BCs and ES-BCs as found in microarray analyses is given for each gene.

Verification of these microarray data with quantitative PCR revealed that out of 11 genes selected from this list 10 genes (*COMT, DYNLT3, NME4, OXCT1, MGMT, PTGR1, MGC3207, CKLF, ZNF167, ZNF626*, and *RPL39L*) were expressed at very low levels or not at all in ES cells and ES-BCs, but were highly upregulated in iPS cells and iPS-BCs (Figure 5, for comparison see microarray expression data in Table 1). Interestingly, all of these 10 iPS/iPS-BC-enriched genes were also strongly expressed in fibroblasts used for reprogramming, their expression levels in fibroblasts being similar to those in iPS cells and iPS-BCs (Figure 5). These data suggest that this group of genes may belong to fibroblast specific-genes that could not be repressed in the process of reprogramming and still remain upregulated in iPS cells. Indeed, two recent studies demonstrated that iPS cells are characterized by a unique gene expression signature, which distinguishes them from ES cells [29,31]. By comparing these data with our list of 47 transcripts upregulated in iPS cells and iPS-BCs we found that 16 of these transcripts (34%) were also detected in these previous studies to be specifically expressed in multiple human iPS cell lines in comparison with various ES cell lines (Table 1). Thus, iPS cell-signature genes appear to be specifically expressed in iPS-BCs but not ES-BCs.

### Gene ontology analysis of genes upregulated in iPS-BCs vs ES-BCs

In order to determine which biological terms are enriched in iPS-BCs compared to ES-BCs, we have performed a functional annotation clustering analysis of 204 unique transcripts that were found to be upregulated in iPS-BCs (see Additional file 4, Table S7). Considering only the significant GO-terms with the p-value < 0.001 this analysis revealed the overrepresentation in iPS-BCs of functional categories related to collagen, extracellular matrix, cell adhesion and tissue development and morphogenesis (Table 2). Genes in these categories included various types of collagens (*COL1A1, COL3A1, COL4A1, COL5A1, COL5A2, COL6A3*) and other extracellular matrix components (*LUM*, *LAMB1, CTGF*), cell surface proteins involved in cell-cell interactions (*CD36, CD44, CD47*) and a variety of transcription factors involved in tissue development and morphogenesis (*HOXA5, HOXB5, SNAI2, NR2F2*). These transcripts were increased in iPS-BCs vs ES-BCs in average by 3,3 ± 2,1 -fold (Additional file 5, Table S11). Since these transcripts are expressed by specific cell types (e.g. collagens by fibroblasts) or involved in specific biological processes (e.g. HOXB5 in lung and gut development), these findings indicate that transcriptional differences between iPS-BCs and ES-BCs may partially occur due to higher abundance (or higher transcriptional activity) of specific non-cardiac cell types in these clusters.

**Table 2:** Functional annotation clustering of 199 DAVID IDs that were upregulated in hiPS-BCs vs hES-BCs.

GO term	Category	No. of genes	p- value	Genes
ECM-receptor interactions	KEGG pathway	12	2.7E-9	CD47, CD36, COL4A1, CD44, COL6A3, COL3A1, COL1A2, RELN, COL1A1, LAMB1, COL5A2, COL5A1

Collagen	Cellular component	9	4.5E-9	COL4A1, LUM, COL6A3, COL3A1, COL1A2, COL12A1, COL1A1, COL5A2, COL5A1

Fibrillar collagen	Cellular component	6	1.4E-7	LUM, COL3A1, COL1A2, COL1A1, COL5A2, COL5A1

Tissue development	Biological process	20	3.3E-4	EDN3, CAV1, PPHLN1, COL3A1, ANXA1, KITLG, POSTN, SNAI2, COL5A2, COL5A1, CD44, HOXA5, CTGF, HOXB5, HLX, PRKAR1A, COL1A2, COL1A1, NR2F2, PITX1

Skin development	Biological process	5	3.6E-4	COL3A1, COL1A2, COL1A1, COL5A2, COL5A1

Focal adhesion	KEGG pathway	10	5.7E-4	CAV1, COL4A1, COL6A3, COL3A1, COL1A2, RELN, COL1A1, LAMB1, COL5A2, COL5A1

GO-terms that were significantly overrepresented (enrichment score > 3, p < 0.001,) and genes included in each category are shown. The GO-terms shown in this table all belong to the same annotation cluster with the enrichment score of 4.56.

### Microarray analysis of human ES cell- and CM-specific and enriched genes

**Figure 6:**
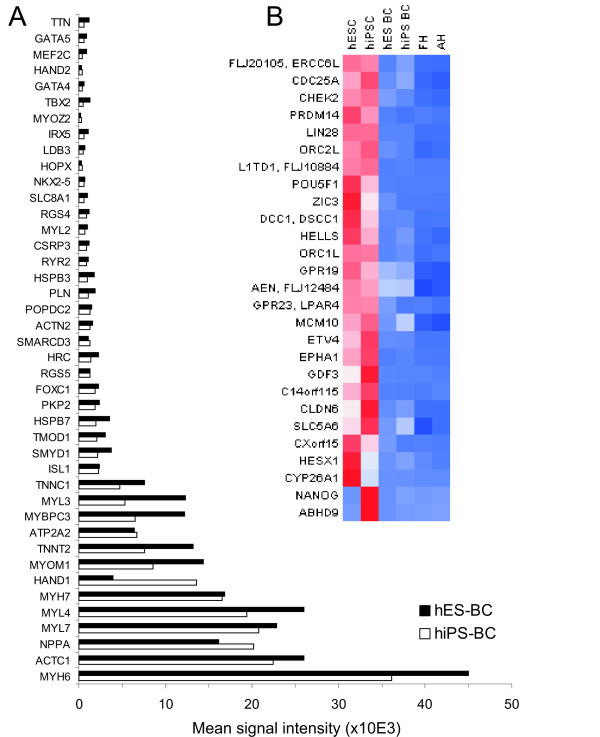
**Expression of cardiac- and ES cell-specific transcripts**. **A**. Comparison of expression levels of 42 known cardiac transcripts in iPS-BCs and ES-BCs. Data are presented as mean gene expression of triplicate samples in relative intensity units. Expression levels of all transcripts were not statistically significant between iPS-BCs and ES-BCs (p > 0,05). For genes, such as *HAND1*, *MYL3 *or *TBX2*, the mean intensity values strongly differ, but the difference is not statistically significant because of high variability in triplicate samples. **B**. Heat map presentation of expression levels of 27 ES cell-specific genes. Among all genes, only the expression of *CYP26A1*, *NANOG *and *ABHD9 *was statistically different between undifferentiated and differentiated iPS and ES cells. Data for additional CM- and ES cell-specific genes are given in the Additional file 6, Tables S12-S14.

We have further compared the expression levels of selected transcripts known to be specifically expressed or enriched in undifferentiated ES cells or CMs. 93.4% of human ES cell-specific or -enriched genes were expressed at similar levels in iPS and ES cells (Figure 6 and Additional file 6, Tables S12 and S13). Furthermore, 89.2% of 83 cardiac specific or enriched genes were expressed at similar levels in iPS-BCs and ES-BCs and out of 34 BC- or CM-associated genes identified in previous studies [32-36] 28 (82.4%) were enriched to a similar extent in both iPS-BCs and ES-BCs in our study (Figure 6 and Additional file 6, Table S14). Further elaboration of these microarray data is given in the Additional file 1, Supplementary results.

Microarray data were validated by comparative qPCR analysis of undifferentiated iPS and ES cells with Human embryonic stem cell 96 StellARray™ qPCR Array (Lonza). This analysis revealed that 82 genes (91.1%) were detected with both methods as being expressed at undistinguishable levels in ES and iPS cells (p > 0.05 and fold change < 2) (Additional file 7, Table S15).

### Comparison of transcriptional profiles between undifferentiated and differentiated iPS and ES cells

**Figure 7:**
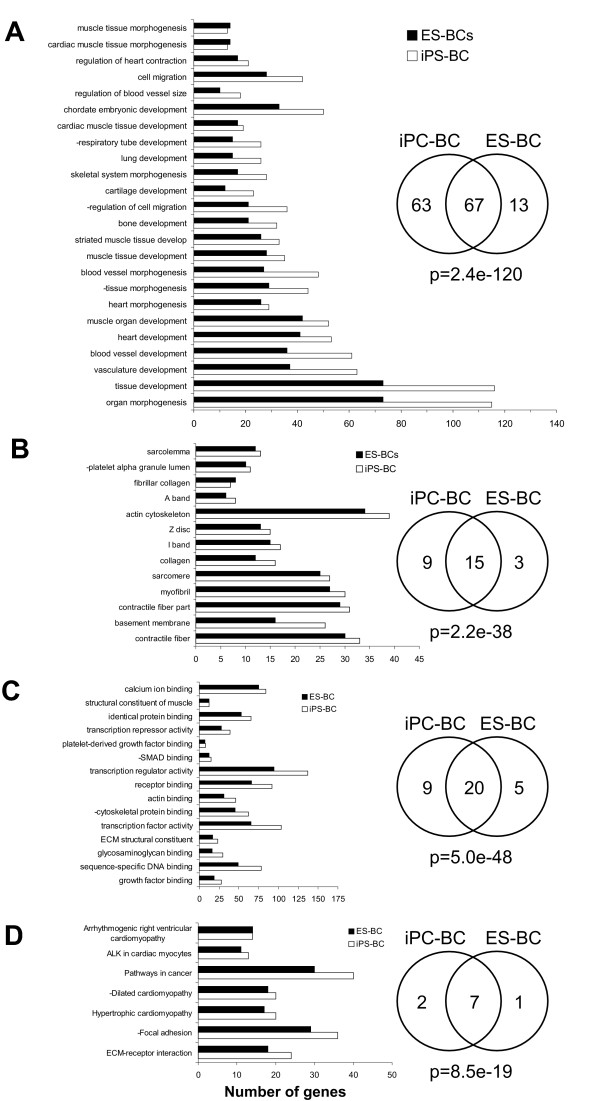
**Gene ontology and KEGG pathway analysis of genes upregulated in beating clusters of ES and iPS cells**. Processes and pathways found to be most overrepresented in microdissected human iPS-BCs and ES-BCs are presented for categories: biological processes (**A**), molecular function **(B)**, cellular pathway (**C) **and KEGG pathways (**D**). Detailed lists of GOterms significantly enriched in iPS-BCs and ES-BCs and the corresponding statistical information are given in Table S20. The p-values under Venn diagrams have been calculated using the Fisher's exact test (we used 17593 as the total number of GO-terms in the GO-database in these calculations).

While transcriptomes of BCs derived from iPS and ES cells were highly similar, as many as 2394 (13.73% of present transcripts) and 2106 probe sets (12.75% of present transcripts) were differentially expressed between undifferentiated iPS cells and iPS-BCs and between undifferentiated ES cells and ES-BCs, respectively (Figure 4D). Among the most upregulated genes in iPS-BCs and ES-BCs were known cardiac genes and the most downregulated genes in both types of BCs belonged to pluripotency markers. More extensive discussion on this subject is provided in the Supplementary results (Additional file 1) and the lists of differentially expressed genes can be found in Additional file 8, Tables S16-S19. Functional annotation analysis was performed in DAVID to identify the most important biological processes that are significantly enriched in iPS- and ES-BCs compared to their undifferentiated counterparts. Taking into account only the GO-terms that had more than 3 genes and p-value < 0,001 this analysis revealed that in categories biological process, molecular function, cellular component and KEGG pathway the overlap between terms enriched in iPS-BCs and ES-BCs was highly significant as determined by Fisher's exact test (Figure 7 and Additional file 9, Table S20). Among the most enriched GO-terms related to cardiomyocytes were heart development, blood vessel and muscle tissue development, myofibril assembly, muscle contraction, ECM structural constituent, calcium ion binding, actin binding, contractile fiber, myofibril, sarcomere, actin cytoskeleton, collagen, Z disc, I band and A band, focal adhesion, dilated, hyperthrophic and arrhythmogenic cardiomyopathy, and ALK pathway in cardiac myocytes (Figure 7 and Additional file 9, Table S20). However, a number of GO-terms were differentially enriched between iPS-BCs and ES-BCs. For example, in the category biological process the iPS-BCs but not ES-BCs were enriched for GO-terms such as kidney development, mesenchyme development, angiogenesis, neural crest cell development, central nervous system development and others, while ES-BCs were enriched, among others, for GO-terms sarcomere organization, muscle thin filament assembly, heart septum morphogenesis and skeletal myofibril assembly (Additional file 9, Table S20). Although these differences may reflect a certain degree of heterogeneity in cellular composition of iPS- and ES-BCs, most categories were enriched in both iPS-BCs and ES-BCs to the same extent, suggesting that they are very similar.

## Discussion

In this study we show that global gene expression profiles of functionally intact CMs and other differentiated cell types enriched in microdissected iPS-BCs and ES-BCs are highly similar. Cardiac specific genes identified in previous reports using enriched [32,33,36] or highly purified human ES-CM-preparations [34,35] were similarly expressed in both iPS-BCs and ES-BCs and only a few percent of transcripts classified as present among all samples were differentially expressed between these two cell populations. The small difference in transcriptomes between iPS-BCs and ES-BCs was not significantly greater than the difference between undifferentiated iPS and ES cells or between different ES cell or different iPS cell lines as reported by others [37-40].

Of interest is the observation that 44.3% of transcripts (47 out of 106) that were upregulated in iPS cells compared to ES cells also remained upregulated in iPS-BCs compared to ES-BCs. Statistical analysis revealed that this overlap is unlikely to occur by chance. Many of these genes were expressed at very high levels in iPS cells and iPS-BCs but were absent from both undifferentiated ES cells and ES-BCs. We could not identify these genes on the list of 530 genes reported to be upregulated in microdissected BCs derived from human ES cell line SA002 by Synnergren and coworkers [32]. However, the significant fraction of these genes (16 out of 47) has been found by other groups to be specifically expressed in various human iPS cell lines compared to ES cell lines [29,31]. In one another comparison, one third of our 47 genes was also found to be overexpressed in at least five different undifferentiated iPS cell lines when compared to several independently derived human ES cell lines (Natalia Polouliakh, Sony Computer Science Laboratories, Tokyo, Japan, personal communication). Thus, these genes may belong to a unique gene expression signature of iPS cells [29,31]. Interestingly, many of these 47 genes have been found to be expressed in foreskin fibroblasts [6] and this was also confirmed by qRT-PCR in our study for 10 randomly selected genes. Thus, this subset of genes may represent fibroblast-associated genes that are expressed in iPS cells because they are refractory to epigenetic silencing during reprogramming, as suggested previously [29,31]. Recent meta-analysis of multiple transcriptional data sets derived from human fibroblasts, ES and iPS cells also revealed that iPS cells share four-times more genes in common with fibroblasts from which they were derived than ES cells and fibroblasts [40].

While the retention of epigenetic memory of somatic cells in undifferentiated iPS cells may explain a subset of differentially expressed genes in iPS vs ES cell comparison, it is more difficult to discern the mechanism behind the highly significant overlap between genes upregulated in iPS vs ES cells and those upregulated in iPS-BC vs ES-BCs. It is unlikely that differential expression of these genes resulted from differential amounts of contaminating murine feeder cells in BCs derived from two types of stem cells, because iPS and ES cells were maintained and differentiated under the same conditions for all three respective biological replicates. Differential contamination of BCs with pluripotent stem cells is also unlikely, because qRT-PCR analyses demonstrated that the expression of endogenous *OCT4 *decreased to a similar extent upon differentiation of both ES and iPS cells, and *SOX2 *could not even be detected in both types of BCs. We have also excluded the possibility that some of the common iPS cell- and iPS-BC-enriched genes are upregulated as a consequence of ectopic reactivation of lentiviral *OCT4 *gene in iPS-BCs, because these genes had not been reported to be the Oct4 targets in previous studies [41-43]. Instead, our analyses indicate that transcriptional differences between iPS-BCs and ES-BCs may, at least partially, be caused by differential cellular composition in these clusters. As revealed by gene ontology analysis, biological terms related to collagen, extracellular matrix, cell adhesion and tissue development were enriched in iPS-BCs compared to ES-BCs by about 3-fold. It remains to be determined whether higher content of fibroblasts in iPS-BCs (or other cell types expressing the overrepresented transcripts) is due to specific properties of the iPS cell line used in this study or due to inherently better capability of different iPS cell lines to differentiate towards cells of their somatic origin. Just recently, two studies using murine iPS cell lines derived from different somatic cell types demonstrated that iPS cells retain some DNA methylation signature characteristic of their somatic tissue of origin and that their cellular origin influences the *in vitro *differentiation propensity of iPS cells [44,45].

The disadvantage of using microdissected beating clusters in our study, which contain CMs and multiple other cell types, is that we are not able to provide a definitive answer to the question whether epigenetic memory of somatic cells may still be retained in some mature iPS cell derivatives. To answer this question, transcriptional profiles of undifferentiated iPS cells and highly purified differentiated cells derived from several iPS cell lines should be compared. We have compared transcriptional profiles of highly purified cardiomyocytes generated by drug selection from a transgenic murine iPS and ES cell line, but could not find any evidence for retention of somatic cell signature in murine iPS-CMs (Fatima A., manuscript in preparation). The same observation has been also recently reported for murine cardiac Nkx2.5-positive progenitor cells isolated by drug selection from several murine iPS and ES cell lines [46]. In this study, the variability in gene expression profiles that was seen between undifferentiated iPS and ES cell lines was even reduced in highly purified lineage-specific cardiac progenitors. Thus, it is unlikely that CMs present in iPS-BCs express any of upregulated genes listed in our Table 1. However, we can not exclude the possibility that other cell types in iPS-BCs still ectopically express some of the genes in this list as a consequence of retention of epigenetic memory of somatic cells. Clearly, additional studies with different cell lines and purified differentiated cell populations will be required to discern between these various possibilities.

A significant progress made in the generation of genetically intact iPS cells [47,48] renders the investigation of consequences of transgene reactivation on the physiology of differentiated cells less relevant. The iPS cell line used in our present report contain sequences of reprogramming transgenes stably integrated into their genome [6] and this may have caused some of the differences in gene expression profiles detected between ES and iPS cells. This notion is supported by observation that iPS cells generated by non-integrating reprogramming methods are transcriptionally more similar to ES cells than those generated with stably integrating viral vectors [40]. Therefore, genetically intact iPS cell lines should be utilized in future studies. However, it is worth mentioning that the genetic integration of viral sequences and incomplete silencing of the lentiviral *OCT4 *transgene in iPS-BCs as observed in our study, and corroborated by Zhang and coworkers [22], apparently did not adversely affect the functional as well as molecular properties of iPS-CMs. This is in agreement with the observation that ectopic Oct4 expression in the heart of adult mice did not lead to any detectable alterations of cell phenotype [49]. Similarly, 303 transcripts differentially expressed between iPS-BC and ES-BCs did not appear to exert a measurable affect on functional properties of CMs contained in iPS-BCs *in vitro*.

## Conclusions

Transcriptional profiles of undifferentiated iPS and ES cells have been extensively analyzed in the past. In this study we provide molecular evidence that not only undifferentiated iPS and ES cells but also their differentiated derivatives contained in spontaneously beating areas have highly similar global transcriptional profiles. Minor differences in gene expression that were identified between iPS and ES cells in undifferentiated and differentiated state did not appear to significantly affect the structural or functional properties of iPS cell-derived cardiomyocytes. However, iPS-BCs appear to retain some of the iPS cell genetic signature partially due to enrichment of specific cell types (such as those expressing collagen and ECM-components) and thus may not be perfectly identical to ES-BCs. These findings emphasize the necessity for detailed functional and molecular analyses of mature iPS cell-derivatives so as to determine the consequences of their expression for the physiology and safety of iPS cell-derivatives.

## Methods

### Culture of undifferentiated human ES and iPS cells

The human iPS cell line derived from foreskin fibroblasts, clone 1 (C1), was kindly provided by James Thomson (University of Wisconsin, Madison, WI, USA) [6]. The informed consent of the patient donating the tissue probe for iPS cell generation was obtained by the original group (see the reference 6). For comparison we used the human ES cell line HES-2 that was generated by the ES Cell International (Singapore, http://www.escellinternational.com/about_esi/index.html) and obtained from the repository at the WiCell Research Institute (Madison, WI, USA, http://www.wicell.org/). The iPS and ES cells were maintained on irradiated murine embryonic fibroblasts (MEFs) in DMEM/F12 medium supplemented with Glutamax, 20% knockout serum replacer, 1% nonessential amino acids (NAA), 0.1 mmol/L β-mercaptoethanol (βME) (Invitrogen, Carlsbad, CA, http://www.invitrogen.com) and with 4 or 100 ng/ml basic fibroblast growth factor (Peprotech, Rocky Hill, NJ, USA, http://www.peprotech.com) for ES cells or iPS cells, respectively. Culture media were changed daily and the cells were passaged by manual dissection of cell clusters every 5-6 days. Work with human ES cells has been approved by the regulatory authorities at the Robert Koch Institute, Berlin, Germany (permission number 1710-79-1-4-2-A10).

### Cardiac differentiation

Cardiac differentiation of human iPS and ES cells was carried out on the murine visceral endoderm-like cell line END2 [15,50]. END2 cell cultures were mitotically inactivated for 3 hours with 10 μg/ml mitomycin C (Sigma-Aldrich) and 1.2 × 10^6 ^cells were plated on 60 mm tissue culture dishes one day before starting the co-culture. To initiate co-cultures, iPS and ES cell colonies were dissociated into clumps by either using collagenase IV (1 mg/ml in DMEM/F-12 at 37°C for 5-10 minutes) or by manual cutting. The differentiation was carried out in Knockout-DMEM having 1 mM Lglutamine, 1% NAA, 0.1 mmol/L (βME) and Penicillin/Streptomycin (100 U/ml and 100 μg/ml, respectively) devoid of serum and serum replacement (all reagents were from Invitrogen). Medium change was performed at day 5, 9, 12 and 15 after initiating the co-cultures.

### RT-PCR and quantitative RT-PCR

Total RNA was isolated using TRIzol Reagent (Invitrogen) from iPS or ES cells and from 40-60 iPS cell-derived BCs (iPS-BCs) or ES cell-derived BCs (ES-BCs) microdissected at day 18 of differentiation. DNase I-treated total RNA (500 ng) was reverse-transcribed using Superscript II RTase (Invitrogen) and random hexamers. cDNA was diluted 1:4 with sterile tri-destilled water and 5 μl were amplified using JumpStart™RedTaq ReadyMix™PCR Reaction Mix (Sigma). Negative controls were generated in RT reactions in which all reaction components were included except RTase. Reactions were terminated at the exponential phase of amplification and products were analyzed by agarose gel electrophoresis. For quantitative RT-PCR the cDNA probes were diluted 1:40 and 2 μl was amplified using SYBR Green PCR Master Mix (Qiagen, Hilden, Germany, http://www.qiagen.com) in triplicate for each sample and each gene. Real-time PCRs were performed in a 7500 Fast System Real Time Cycler (Applied Biosystems, Foster City, CA, USA, http://www.appliedbiosystems.com) and analyzed with SDSShell 1.4 software (Applied Biosystems). *GAPDH *was used for normalization of expression levels of individual genes. Primers used are listed in Additional file 1, Table S1. Microarray results obtained for undifferentiated iPS and ES cells were validated using Human Embryonic Stem Cell 96 StellARray™ qPCR array (Lonza, Cologne, Germany, http://www.lonza.com) (see Supplementary methods in Additional file 1).

### Immuncytochemistry

iPS-BCs and ES-BCs were microdissected on day 15 of differentiation and plated on fibronectin-coated (2 μg/ml) μ-dishes^35 mm, low ^(Ibidi GmbH, Munich, Germany, http://www.ibidi.de). Three to five days after plating, the cells were fixed with ice-cold methanol at -20°C. After washing with phosphate buffered saline (PBS, pH 7.4), the cells were blocked for one hour with 5% bovine serum albumin (BSA) in PBS.

Incubation with anti-sarcomeric actinin (Sigma-Aldrich, clone EA-53, 1:400 dilution) and anti-cardiac troponin T (cTnT) (Neomarkers, Fremont, CA, clone 13-11, 1:100 dilution) was performed in 1% BSA overnight at 4°C. After washing with PBS the samples were incubated with secondary AlexaFluor555-conjugated antibodies (Invitrogen) for 90 minutes at ambient temperature. Nuclei were counterstained with Hoechst 33432 (2 μg/ml). Samples were embedded in ProLong Gold antifade reagent (Invitrogen) and observed on Axiovert Microscope (Carl-Zeiss, Jena, Germany, http://www.zeiss.de) equipped with the image processing software Axiovision 4.5.

### Multi-Electrode Array (MEA) measurements

To characterize the functional properties of iPS-CMs and ES-CMs, extracellular recordings of field potentials (FPs) were performed using a microelectrode array (MEA) data acquisition system (Multi Channel Systems, Reutlingen, Germany, http://www.multichannelsystems.com) as described previously [17,51]. For this purpose, iPS- and ES-BCs were microdissected on day 15 of differentiation, plated on fibronectin-coated (2 μg/ml) MEA culture plates in DMEM supplemented with 20% fetal bovine serum (FBS), 1% NAA and 0.1 mmol/L βME and measured 24-48 hours after attachment. Standard measurements were performed at 5 kHz in serum-free IMDM. Isoproterenol (Sigma-Aldrich), a standard stimulator of the β-adrenergic signaling cascade, and carbachol (Sigma-Aldrich), a synthetic acetylcholine analogon, were dissolved in serum-free medium. During recordings, the temperature was kept at 37°C. Data were analyzed off-line with MATLAB (The Mathworks, Natick, MA, USA, http://www.mathworks.com) [52].

### Measurement of intracellular Ca^2+^

Ca^2+ ^imaging experiments were performed as described previously [53]. Briefly, BCs were isolated on day 18 of differentiation and loaded with 2.5 μM Fura-2 AM (Molecular Probes, Eugene, OR, USA, http://www.molecularprobes.com) and 0.02% Pluronic-127 (Sigma-Aldrich) in modified Tyrode solution containing (in mmol/l): NaCl 140, KCl 5.4, MgCl_2 _1, sodium pyruvate 2, CaCl_2 _2, HEPES 10, glucose 10 (pH 7.4) for 30 minutes at room temperature [54]. Following the loading procedure the BCs were washed and incubated for 30 minutes at 37°C for deesterification before measurements. After alternating monochromatic excitation with 340 nm and 380 nm, emitted light was detected using a charge-coupled device cooled camera (TILL IMAGO CCD, TILL Photonics, Planegg, Germany, http://www.till-photonics.com) with a sampling rate of 40 Hz. The cell aggregates were perfused with Tyrode solution at 1 ml/min. Two perfusion systems were used: chamber perfusion with approx. 1 cm distance to the clusters and 'puff'-perfusion placed directly in front of the cells. The latter was used for caffeine (10 mmol/L) application in order to estimate the sarcoplasmic reticulum (SR) Ca^2+ ^load. Ratio F340/F380 data were background subtracted (TILLvisION, TILL Photonics) and transient parameters were analyzed with Chart software v5 (ADInstruments, Castle Hill, Australia, http://www.adinstruments.com). Results are expressed as mean ± SEM. Statistical analysis was performed by Student's t-test or paired t-test (p-value < 0.05 was considered significant).

### Microarray procedure and analysis

Total RNA was extracted from undifferentiated ES and iPS cells and their corresponding BCs microdissected at day 18 of differentiation using the TRIzol reagent. Human RNA from fetal (FH) and adult hearts (AH) was purchased from Clontech (Saint-Germain-en-Laye, France, http://www.clontech-europe.com/). The characteristics of these samples are summarized in Additional file 1, Table S5. Biotinlabeled cRNA preparation for the Illumina platform was performed using the Ambion^® ^Illumina RNA amplification kit (Ambion Europe, Huntington, Cambridgeshire, UK). Afterwards, biotin-labeled cRNA (750 ng) was hybridized to Sentrix^® ^whole genome bead chips comprising 48803 probe sets (Human HT-12_V3, Illumina, San Diego, CA, USA, http://www.illumina.com/) and scanned on the Illumina^® ^BeadStation 500×. Across all samples, 16837 ± 1311 probe sets (34.5 ± 2.7%) received present calls as defined by the detection p-value of < 0.05 (n = 3). Raw data extraction of mRNA microarrays was performed with Beadstudio 3.1.1.0 software using the Beadstudio Gene Expression Analysis Module 3.1.8. All further analysis was performed in R (http://www.r-project.org, version 2.8.0) using Bioconductor packages. For further analysis we used quantile normalization implemented in the affy package. Variable genes were defined by a coefficient of variation (SD/mean) between 0.5-10. Determination of present calls was based on the detection p-value assessed by Beadstudio software; a gene was called present if the detection p-value was < 0.05. Otherwise the mRNA transcript was called absent. Differentially expressed genes were selected using a fold change/p-value filter with the following criteria: only p-values smaller than 0.05 and an expression change higher than 2 fold and a difference between mean intensity signals greater than 100 were considered statistically significant for further analysis. The Benjamini-Hochberg method was used to adjust the raw p-values to control the false discovery rate. The fold-change was calculated by dividing the mean intensity of the genes in one group by that in the other group. If this number was less than one, the negative reciprocal was used. Hierarchical cluster analysis was performed using the hcluster method in R. Before clustering, the data were log2 transformed. Distances of the samples were calculated using Pearson correlation and clusters were formed by taking the average of each cluster. Principal component (PC) analysis was performed using the pcurve package in R. When visualizing PC analysis results, the first 3 principal components (coordinates) were z-transformed (mean = 0, standard deviation = 1) and subsequently plotted in 3 D. The first PC axis accounted for 63.8% of the variance in the data set of variable transcripts, while the second and third PC axes accounted for 20.9% and 0.07% of the variance, respectively All heat maps were visualized using MAYDAY [55]. The enrichment of specific biological processes, molecular functions, cellular components and pathways among differentially expressed genes was analyzed using the Database for Annotation, Visualization and Integrated Discovery (DAVID, http://david.abcc.ncifcrf.gov/) bioinformatics resource [56,57] and functional categories and biological pathways annotated by the Gene ontology tool [58] and the Kyoto Encyclopedia of Genes and Genomes (KEGG) pathway database [59]. Annotations were considered significantly overrepresented when the p-value of the Fisher's exact test as used by DAVID was < 0.001 (enrichment score in functional annotation clustering > 3). Fisher's exact test was performed using an online calculator at http://www.langsrud.com/fisher.htm.

## Abbreviations

CM: cardiomyocytes; iPS: induced pluripotent stem (cells); ES: embryonic stem (cells); iPS-CM: induced pluripotent stem cell-derived cardiomyocytes; ES-CM: embryonic stem cell-derived cardiomyocytes; BC: beating clusters; iPS-BC: induced pluripotent stem cell-derived beating clusters; ES-BC: embryonic stem cell-derived beating cluster; cTnT: cardiac troponin T; MEA: microelectrode arrays; ISO: isoproterenol; CCh: carbachol; FP: field potential; PCA: principal component analysis; FH: fetal heart; AH: adult heart; PBS: phosphate buffered saline; BSA: bovine serum albumin.

## Authors' contributions

MKG and DJI: collection and assembly of data, data analysis and interpretation, manuscript writing; AG, MM, FN: collection and assembly of data, data analysis and interpretation; KP, HL, and SC: collection and assembly of data; MR: data analysis and interpretation, JLS: financial support, manuscript writing; JH: financial support, final approval of manuscript; TŠ: conception and design, financial support, assembly of data, data analysis and interpretation, manuscript writing, final approval of manuscript. All authors read and approved the final manuscript.

## Supplementary Material

Additional file 1 **Collection of Figures S1-S3, Tables S1-S5, Supplemental results, methods and references**. This file is a PDF file containing Figure S1 (characterization of pluripotency markers in undifferentiated human iPS and ES cells), Figure S2 (assessment of the cardiogenic potential of human iPS and ES cells), Figure S3 (expression of pluripotency genes in beating clusters derived from human iPS and ES cells), Table S1 (primers used for RT-PCR analyses), Table S2 (statistical analysis of MEA measurements), Table S3 (detailed analysis of spontaneous Ca2+ transients), Table S4 (coupling between contractions and Ca2+ transients), Table S5 (description of samples used for transcriptional profiling), and Supplementary results, methods and references. Click here for file

Additional file 2 **List of genes that are differentially expressed across all samples**. This file is an Excel file containing Table S6 with the list of 4254 variable genes and their expression levels in all samples. Variable genes were defined by a coefficient of variation (SD/mean) between 0.5-10. Click here for file

Additional file 3 **List of genes differentially expressed between undifferentiated iPS and ES cells**. This Excel file contains Tables S7 and S8 with the list of genes that are, respectively, upregulated and downregulated in undifferentiated iPS cells compared to ES cells. Click here for file

Additional file 4 **List of genes differentially expressed between iPS cell- and ES cell-derived beating clusters (BCs)**. This Excel file contains Tables S9 and S10 with the list of genes that are, respectively, upregulated and downregulated in iPS-BCs compared to ES-BCs. Click here for file

Additional file 5 **Expression levels of selected genes included in GO-terms that were found to be overrepresented in iPS-BCs compared to ES-BCs**. This PDF file contains the Table S11 displaying the expression levels of selected fibroblastassociated genes found to be overexpressed in iPS-BCs compared to ES-BCs. Click here for file

Additional file 6 **Expression levels of selected human ES cell- and cardiacspecific or enriched genes across all samples**. This is a PDF file in a landscape format containing Table S12 (list of 27 ES cell-specific genes and their expression levels across all samples), Table S13 (list of 49 human ES cell-enriched genes with their expression levels in all samples) and Table S14 (list of 83 cardiac specific or enriched genes and their expression levels across all samples). Click here for file

Additional file 7 **qRT-PCR validation of microarray data with Human ES cell StellArray qPCR Array (Lonza)**. This file is a PDF document containing Table S15 showing the results of qRT-PCR analysis of expression of selected pluripotency and differentiations genes in undifferentiated human ES and iPS cells in comparison with microarray data obtained in this study. Click here for file

Additional file 8 **List of differentially expressed genes in iPS-BC versus iPS cell comparison and ES-BC versus ES cell comparison**. This file is an Excel file containing Table S16 (list of 1235 genes upregulated in iPS-BC compared to iPS cells), Table S17 (list of 1014 genes downregulated in iPS-BC compared to iPS cells), Table S18 (list of 875 genes upregulated in ES-BC compared to ES cells), and Table S19 (list of 1103 genes downregulated in ES-BC compared to ES cells). Click here for file

Additional file 9 **Gene ontology analysis of genes upregulated in iPS-BCs and ES-BCs as compared to their respective undifferentiated counterparts**. This file is a PDF document containing Table S20 with the list of all overrepresented GO terms in iPS-BCs compared to iPS cells and in ES-BCs compared to ES cells. Click here for file

## References

[B1] Takahashi K Yamanaka S Induction of pluripotent stem cells from mouse embryonic and adult fibroblast cultures by defined factors Cell 2006 126 4 663 676 10.1016/j.cell.2006.07.024 16904174

[B2] Meissner A Wernig M Jaenisch R Direct reprogramming of genetically unmodified fibroblasts into pluripotent stem cells Nat Biotechnol 2007 25 10 1177 1181 10.1038/nbt1335 17724450

[B3] Okita K Ichisaka T Yamanaka S Generation of germline-competent induced pluripotent stem cells Nature 2007 448 7151 313 317 10.1038/nature05934 17554338

[B4] Okita K Nakagawa M Hyenjong H Ichisaka T Yamanaka S Generation of mouse induced pluripotent stem cells without viral vectors Science 2008 322 5903 949 953 10.1126/science.1164270 18845712

[B5] Wernig M Meissner A Foreman R Brambrink T Ku M Hochedlinger K Bernstein BE Jaenisch R In vitro reprogramming of fibroblasts into a pluripotent ES-cell-like state Nature 2007 448 7151 318 324 10.1038/nature05944 17554336

[B6] Yu J Vodyanik MA Smuga-Otto K Antosiewicz-Bourget J Frane JL Tian S Nie J Jonsdottir GA Ruotti V Stewart R Induced pluripotent stem cell lines derived from human somatic cells Science 2007 318 5858 1917 1920 10.1126/science.1151526 18029452

[B7] Schenke-Layland K Rhodes KE Angelis E Butylkova Y Heydarkhan-Hagvall S Gekas C Zhang R Goldhaber JI Mikkola HK Plath K Reprogrammed mouse fibroblasts differentiate into cells of the cardiovascular and hematopoietic lineages Stem Cells 2008 26 6 1537 1546 10.1634/stemcells.2008-0033 18450826 PMC2873147

[B8] Sonja S Haruta M Matsunaga Y Fukushima S Ikeda T Takahashi K Yamanaka S Nishimura Y Characterization of dendritic cells and macrophages generated by directed differentiation from mouse induced pluripotent stem cells Stem Cells 2009 27 5 1021 1031 10.1002/stem.33 19415766

[B9] Tateishi K He J Taranova O Liang G D'Alessio AC Zhang Y Generation of insulin-secreting islet-like clusters from human skin fibroblasts J Biol Chem 2008 283 46 31601 31607 10.1074/jbc.M806597200 18782754

[B10] Taura D Noguchi M Sone M Hosoda K Mori E Okada Y Takahashi K Homma K Oyamada N Inuzuka M Adipogenic differentiation of human induced pluripotent stem cells: comparison with that of human embryonic stem cells FEBS Lett 2009 583 6 1029 1033 10.1016/j.febslet.2009.02.031 19250937

[B11] Xie CQ Huang H Wei S Song LS Zhang J Ritchie RP Chen L Zhang M Chen YE A comparison of murine smooth muscle cells generated from embryonic versus induced pluripotent stem cells Stem Cells Dev 2009 18 5 741 748 10.1089/scd.2008.0179 18795840 PMC2914231

[B12] Zhang D Jiang W Liu M Sui X Yin X Chen S Shi Y Deng H Highly efficient differentiation of human ES cells and iPS cells into mature pancreatic insulin-producing cells Cell Res 2009 19 4 429 438 10.1038/cr.2009.28 19255591

[B13] Kehat I Kenyagin-Karsenti D Snir M Segev H Amit M Gepstein A Livne E Binah O Itskovitz-Eldor J Gepstein L Human embryonic stem cells can differentiate into myocytes with structural and functional properties of cardiomyocytes J Clin Invest 2001 108 3 407 414 11489934 10.1172/JCI12131 PMC209357

[B14] He JQ Ma Y Lee Y Thomson JA Kamp TJ Human embryonic stem cells develop into multiple types of cardiac myocytes: action potential characterization Circ Res 2003 93 1 32 39 10.1161/01.RES.0000080317.92718.99 12791707

[B15] Mummery C Ward-van Oostwaard D Doevendans P Spijker R van den Brink S Hassink R van der Heyden M Opthof T Pera M de la Riviere AB Differentiation of human embryonic stem cells to cardiomyocytes: role of coculture with visceral endoderm-like cells Circulation 2003 107 21 2733 2740 10.1161/01.CIR.0000068356.38592.68 12742992

[B16] Kuzmenkin A Liang H Xu G Pfannkuche K Eichhorn H Fatima A Luo H Saric T Wernig M Jaenisch R Functional characterization of cardiomyocytes derived from murine induced pluripotent stem cells in vitro Faseb J 2009 19703934 10.1096/fj.08-128546

[B17] Mauritz C Schwanke K Reppel M Neef S Katsirntaki K Maier LS Nguemo F Menke S Haustein M Hescheler J Generation of functional murine cardiac myocytes from induced pluripotent stem cells Circulation 2008 118 5 507 517 10.1161/CIRCULATIONAHA.108.778795 18625890

[B18] Narazaki G Uosaki H Teranishi M Okita K Kim B Matsuoka S Yamanaka S Yamashita JK Directed and systematic differentiation of cardiovascular cells from mouse induced pluripotent stem cells Circulation 2008 118 5 498 506 10.1161/CIRCULATIONAHA.108.769562 18625891

[B19] Pfannkuche K Liang H Hannes T Xi J Fatima A Nguemo F Matzkies M Wernig M Jaenisch R Pillekamp F Cardiac myocytes derived from murine reprogrammed fibroblasts: intact hormonal regulation, cardiac ion channel expression and development of contractility Cell Physiol Biochem 2009 24 12 73 86 10.1159/000227815 19590195

[B20] Tanaka T Tohyama S Murata M Nomura F Kaneko T Chen H Hattori F Egashira T Seki T Ohno Y In vitro pharmacologic testing using human induced pluripotent stem cell-derived cardiomyocytes Biochem Biophys Res Commun 2009 385 4 497 502 10.1016/j.bbrc.2009.05.073 19464263

[B21] Yokoo N Baba S Kaichi S Niwa A Mima T Doi H Yamanaka S Nakahata T Heike T The effects of cardioactive drugs on cardiomyocytes derived from human induced pluripotent stem cells Biochem Biophys Res Commun 2009 387 3 482 488 10.1016/j.bbrc.2009.07.052 19615974

[B22] Zhang J Wilson GF Soerens AG Koonce CH Yu J Palecek SP Thomson JA Kamp TJ Functional cardiomyocytes derived from human induced pluripotent stem cells Circ Res 2009 104 4 e30 41 10.1161/CIRCRESAHA.108.192237 19213953 PMC2741334

[B23] Gai H Leung EL Costantino PD Aguila JR Nguyen DM Fink LM Ward DC Ma Y Generation and characterization of functional cardiomyocytes using induced pluripotent stem cells derived from human fibroblasts Cell Biol Int 2009 33 11 1184 1193 10.1016/j.cellbi.2009.08.008 19729070

[B24] Zwi L Caspi O Arbel G Huber I Gepstein A Park IH Gepstein L Cardiomyocyte differentiation of human induced pluripotent stem cells Circulation 2009 120 15 1513 1523 10.1161/CIRCULATIONAHA.109.868885 19786631

[B25] Haase A Olmer R Schwanke K Wunderlich S Merkert S Hess C Zweigerdt R Gruh I Meyer J Wagner S Generation of induced pluripotent stem cells from human cord blood Cell Stem Cell 2009 5 4 434 441 10.1016/j.stem.2009.08.021 19796623

[B26] Germanguz I Sedan O Zeevi-Levin N Shtreichman R Barak E Ziskind A Eliyahu S Meiry G Amit M Itskovitz-Eldor J Molecular characterization and functional properties of cardiomyocytes derived from human inducible pluripotent stem cells J Cell Mol Med 2009 in press 20041972 10.1111/j.1582-4934.2009.00996.x PMC3822492

[B27] Lowry WE Richter L Yachechko R Pyle AD Tchieu J Sridharan R Clark AT Plath K Generation of human induced pluripotent stem cells from dermal fibroblasts Proc Natl Acad Sci USA 2008 105 8 2883 2888 10.1073/pnas.0711983105 18287077 PMC2268554

[B28] Maherali N Sridharan R Xie W Utikal J Eminli S Arnold K Stadtfeld M Yachechko R Tchieu J Jaenisch R Directly reprogrammed fibroblasts show global epigenetic remodeling and widespread tissue contribution Cell Stem Cell 2007 1 1 55 70 10.1016/j.stem.2007.05.014 18371336

[B29] Chin MH Mason MJ Xie W Volinia S Singer M Peterson C Ambartsumyan G Aimiuwu O Richter L Zhang J Induced pluripotent stem cells and embryonic stem cells are distinguished by gene expression signatures Cell Stem Cell 2009 5 1 111 123 10.1016/j.stem.2009.06.008 19570518 PMC3448781

[B30] Doi A Park IH Wen B Murakami P Aryee MJ Irizarry R Herb B Ladd- Acosta C Rho J Loewer S Differential methylation of tissue- and cancer-specific CpG island shores distinguishes human induced pluripotent stem cells, embryonic stem cells and fibroblasts Nat Genet 2009 41 12 1350 1353 10.1038/ng.471 19881528 PMC2958040

[B31] Marchetto MC Yeo GW Kainohana O Marsala M Gage FH Muotri AR Transcriptional signature and memory retention of human-induced pluripotent stem cells PLoS One 2009 4 9 e7076 10.1371/journal.pone.0007076 19763270 PMC2741600

[B32] Synnergren J Akesson K Dahlenborg K Vidarsson H Ameen C Steel D Lindahl A Olsson B Sartipy P Molecular signature of cardiomyocyte clusters derived from human embryonic stem cells Stem Cells 2008 26 7 1831 1840 10.1634/stemcells.2007-1033 18436862

[B33] Cao F Wagner RA Wilson KD Xie X Fu JD Drukker M Lee A Li RA Gambhir SS Weissman IL Transcriptional and functional profiling of human embryonic stem cell-derived cardiomyocytes PLoS ONE 2008 3 10 e3474 10.1371/journal.pone.0003474 18941512 PMC2565131

[B34] Xu XQ Soo SY Sun W Zweigerdt R Global expression profile of highly enriched cardiomyocytes derived from human embryonic stem cells Stem Cells 2009 27 9 2163 2174 10.1002/stem.166 19658189

[B35] Kita-Matsuo H Barcova M Prigozhina N Salomonis N Wei K Jacot JG Nelson B Spiering S Haverslag R Kim C Lentiviral vectors and protocols for creation of stable hESC lines for fluorescent tracking and drug resistance selection of cardiomyocytes PLoS One 2009 4 4 e5046 10.1371/journal.pone.0005046 19352491 PMC2662416

[B36] Beqqali A Kloots J Ward-van Oostwaard D Mummery C Passier R Genome-wide transcriptional profiling of human embryonic stem cells differentiating to cardiomyocytes Stem Cells 2006 24 8 1956 1967 10.1634/stemcells.2006-0054 16675594

[B37] Moore JC Fu J Chan YC Lin D Tran H Tse HF Li RA Distinct cardiogenic preferences of two human embryonic stem cell (hESC) lines are imprinted in their proteomes in the pluripotent state Biochem Biophys Res Commun 2008 372 4 553 558 10.1016/j.bbrc.2008.05.076 18503758 PMC2665880

[B38] Osafune K Caron L Borowiak M Martinez RJ Fitz-Gerald CS Sato Y Cowan CA Chien KR Melton DA Marked differences in differentiation propensity among human embryonic stem cell lines Nat Biotechnol 2008 26 3 313 315 10.1038/nbt1383 18278034

[B39] Adewumi O Aflatoonian B Ahrlund-Richter L Amit M Andrews PW Beighton G Bello PA Benvenisty N Berry LS Bevan S Characterization of human embryonic stem cell lines by the International Stem Cell Initiative Nat Biotechnol 2007 25 7 803 816 10.1038/nbt1318 17572666

[B40] Wang Y Mah N Prigione A Wolfrum K Andrade-Navarro MA Adjaye J A transcriptional roadmap to the induction of pluripotency in somatic cells Stem Cell Rev 2010 6 2 282 296 10.1007/s12015-010-9137-2 20336394

[B41] Campbell PA Perez-Iratxeta C Andrade-Navarro MA Rudnicki MA Oct4 targets regulatory nodes to modulate stem cell function PLoS One 2007 2 6 e553 10.1371/journal.pone.0000553 17579724 PMC1891092

[B42] Matoba R Niwa H Masui S Ohtsuka S Carter MG Sharov AA Ko MS Dissecting Oct3/4-regulated gene networks in embryonic stem cells by expression profiling PLoS One 2006 1 e26 10.1371/journal.pone.0000026 17183653 PMC1762406

[B43] Sharov AA Masui S Sharova LV Piao Y Aiba K Matoba R Xin L Niwa H Ko MS Identification of Pou5f1, Sox2, and Nanog downstream target genes with statistical confidence by applying a novel algorithm to time course microarray and genome-wide chromatin immunoprecipitation data BMC Genomics 2008 9 269 10.1186/1471-2164-9-269 18522731 PMC2424064

[B44] Kim K Doi A Wen B Ng K Zhao R Cahan P Kim J Aryee MJ Ji H Epigenetic memory in induced pluripotent stem cells Nature 2010 in press 10.1038/nature09342 PMC3150836 20644535

[B45] Polo JM Liu S Figueroa ME Kulalert W Eminli S Tan KY Apostolou E Stadtfeld M Li Y Cell type of origin influences the molecular and functional properties of mouse induced pluripotent stem cells Nature Biotechnology 2010 in press 20644536 10.1038/nbt.1667 PMC3148605

[B46] van Laake LW Qian L Cheng P Huang Y Hsiao EC Conklin B Srivastava D Reporter-Based Isolation of Induced Pluripotent Stem Cell-and Embryonic Stem Cell-Derived Cardiac Progenitors Reveals Limited Gene Expression Variance Circ Res 2010 in press 20558827 10.1161/CIRCRESAHA.109.215434 PMC2919280

[B47] Kim D Kim CH Moon JI Chung YG Chang MY Han BS Ko S Yang E Cha KY Lanza R Generation of human induced pluripotent stem cells by direct delivery of reprogramming proteins Cell Stem Cell 2009 4 6 472 476 10.1016/j.stem.2009.05.005 19481515 PMC2705327

[B48] Zhou H Wu S Joo JY Zhu S Han DW Lin T Trauger S Bien G Yao S Zhu Y Generation of induced pluripotent stem cells using recombinant proteins Cell Stem Cell 2009 4 5 381 384 10.1016/j.stem.2009.04.005 19398399 PMC10182564

[B49] Hochedlinger K Yamada Y Beard C Jaenisch R Ectopic expression of Oct- 4 blocks progenitor-cell differentiation and causes dysplasia in epithelial tissues Cell 2005 121 3 465 477 10.1016/j.cell.2005.02.018 15882627

[B50] Xu XQ Graichen R Soo SY Balakrishnan T Rahmat SN Sieh S Tham SC Freund C Moore J Mummery C Chemically defined medium supporting cardiomyocyte differentiation of human embryonic stem cells Differentiation 2008 76 9 958 970 18557764 10.1111/j.1432-0436.2008.00284.x

[B51] Reppel M Boettinger C Hescheler J Beta-adrenergic and muscarinic modulation of human embryonic stem cell-derived cardiomyocytes Cell Physiol Biochem 2004 14 46 187 196 10.1159/000080326 15319521

[B52] Egert U Knott T Schwarz C Nawrot M Brandt A Rotter S Diesmann M MEA-Tools: an open source toolbox for the analysis of multi-electrode data with MATLAB J Neurosci Methods 2002 117 1 33 42 10.1016/S0165-0270(02)00045-6 12084562

[B53] Kolossov E Fleischmann BK Liu Q Bloch W Viatchenko-Karpinski S Manzke O Ji GJ Bohlen H Addicks K Hescheler J Functional characteristics of ES cell-derived cardiac precursor cells identified by tissue-specific expression of the green fluorescent protein J Cell Biol 1998 143 7 2045 2056 10.1083/jcb.143.7.2045 9864374 PMC2175221

[B54] Pillekamp F Reppel M Rubenchyk O Pfannkuche K Matzkies M Bloch W Sreeram N Brockmeier K Hescheler J Force measurements of human embryonic stem cell-derived cardiomyocytes in an in vitro transplantation model Stem Cells 2007 25 1 174 180 10.1634/stemcells.2006-0094 16973834

[B55] Dietzsch J Gehlenborg N Nieselt K Mayday-a microarray data analysis workbench Bioinformatics 2006 22 8 1010 1012 10.1093/bioinformatics/btl070 16500939

[B56] Dennis G Jr Sherman BT Hosack DA Yang J Gao W Lane HC Lempicki RA DAVID: Database for Annotation, Visualization, and Integrated Discovery Genome Biol 2003 4 5 P3 10.1186/gb-2003-4-5-p3 12734009 PMC193660

[B57] Huang da W Sherman BT Lempicki RA Systematic and integrative analysis of large gene lists using DAVID bioinformatics resources Nat Protoc 2009 4 1 44 57 10.1038/nprot.2008.211 19131956

[B58] Ashburner M Ball CA Blake JA Botstein D Butler H Cherry JM Davis AP Dolinski K Dwight SS Eppig JT Gene ontology: tool for the unification of biology. The Gene Ontology Consortium Nat Genet 2000 25 1 25 29 10.1038/75556 10802651 PMC3037419

[B59] Kanehisa M Goto S KEGG: kyoto encyclopedia of genes and genomes Nucleic Acids Res 2000 28 1 27 30 10.1093/nar/28.1.27 10592173 PMC102409

