# Supplementary material for: Global transcriptional profiles of beating clusters derived from human induced pluripotent stem cells and embryonic stem cells are highly similar
Source: BMC Dev Biol. 2010 Sep 15;10:98. doi: 10.1186/1471-213X-10-98 (PMC2946283; doi:10.1186/1471-213X-10-98)
Supplement: Additional file 1 — Collection of Figures S1-S3, Tables S1-S5, Supplemental results, methods and references. This file is a PDF file containing Figure S1 (characterization of pluripotency markers in undifferentiated human iPS and ES cells), Figure S2 (assessment of the cardiogenic potential of human iPS and ES cells), Figure S3 (expression of pluripotency genes in beating clusters derived from human iPS and ES cells), Table S1 (primers used for RT-PCR analyses), Table S2 (statistical analysis of MEA measurements), Table S3 (detailed analysis of spontaneous Ca2+ transients), Table S4 (coupling between contractions and Ca2+ transients), Table S5 (description of samples used for transcriptional profiling), and Supplementary results, methods and references. [file 1471-213X-10-98-S1.PDF]

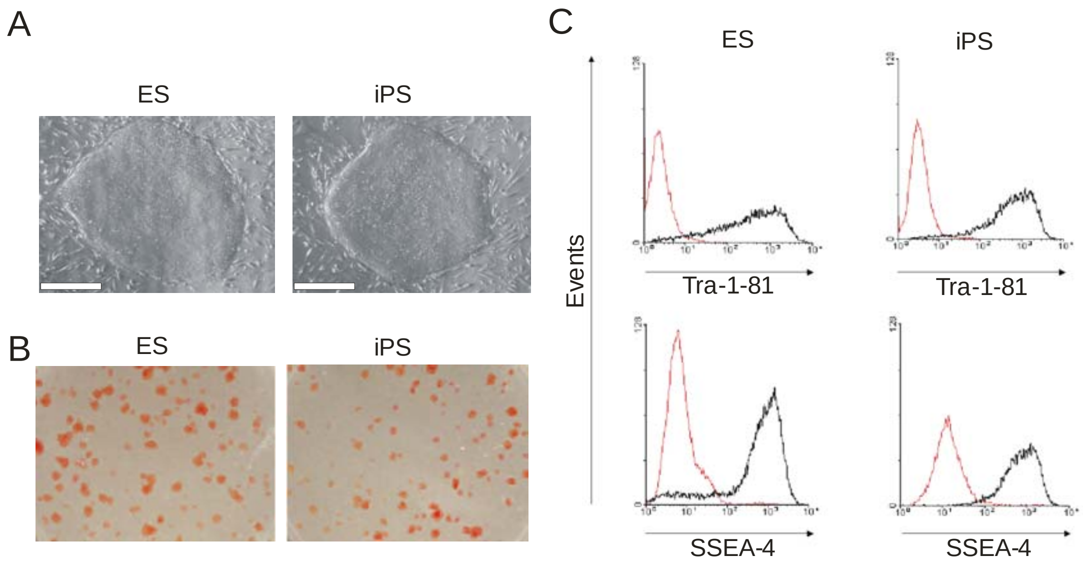

Figure S1 - Characterization of pluripotency markers in undifferentiated human iPS and ES cells. Human iPS (Foreskin C1) and ES cell (HES2) cultures that were used for cardiac differentiations were composed of compact flat colonies, which exhibited well-defined borders (A), stained positively for alkaline phosphatase (B), expressed comparable levels of ES cell markers SSEA-4 and Tra-1-81 (C) and formed teratoma upon injection into immunodeficient mice (data not shown). Isotype control antibodies were used as controls in flow cytometric analyses in panel C and are shown in red.

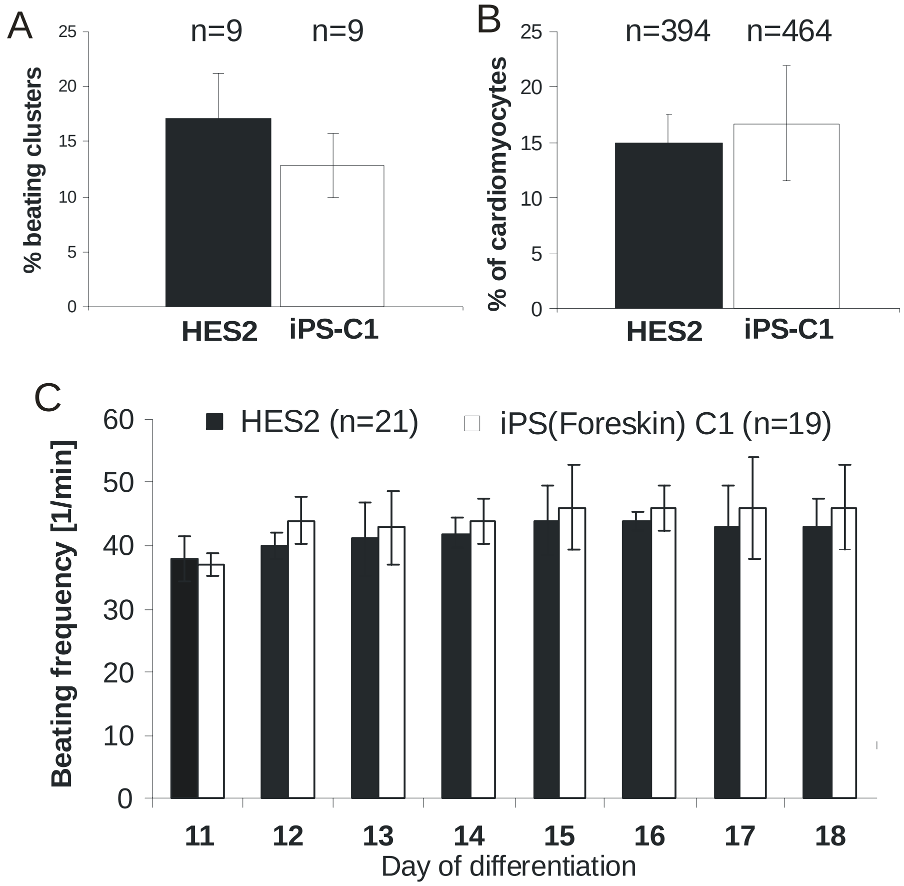

Figure S2 - Assessment of the cardiogenic potential of human iPS and ES cells. A. Human ES cells HES-2 and iPS cells iPS(Foreskin) C1 were differentiated on END2 feeder cells. Contracting cardiac outgrowths were first observed on day 11 of differentiation in both cell types and these contractions were stable for up to three months. The percentage of beating clusters (BCs) was determined on day 15 of differentiation (n=9; three plates/experiment in three independent differentiations). The difference in the yields is not statistically significant as determined with the Student t-test (p=0.128). **B.** Fraction of cardiomyocytes in BCs of human ES and iPS cells. BCs were microdissected, enzymatically dissociated and cells were plated on fibronectin-coated plates. The percentage of α-actinin positive cells was determined by scoring of 394 ES and 464 iPS cell-derived cells. **C.** Beating frequency of human iPS and ES cell-derived BCs were scored on a microscope with heated plate holder (37°C) in three independent differentiations for each time point (n=19 iPS-BCs and n=21 ES-BCs).

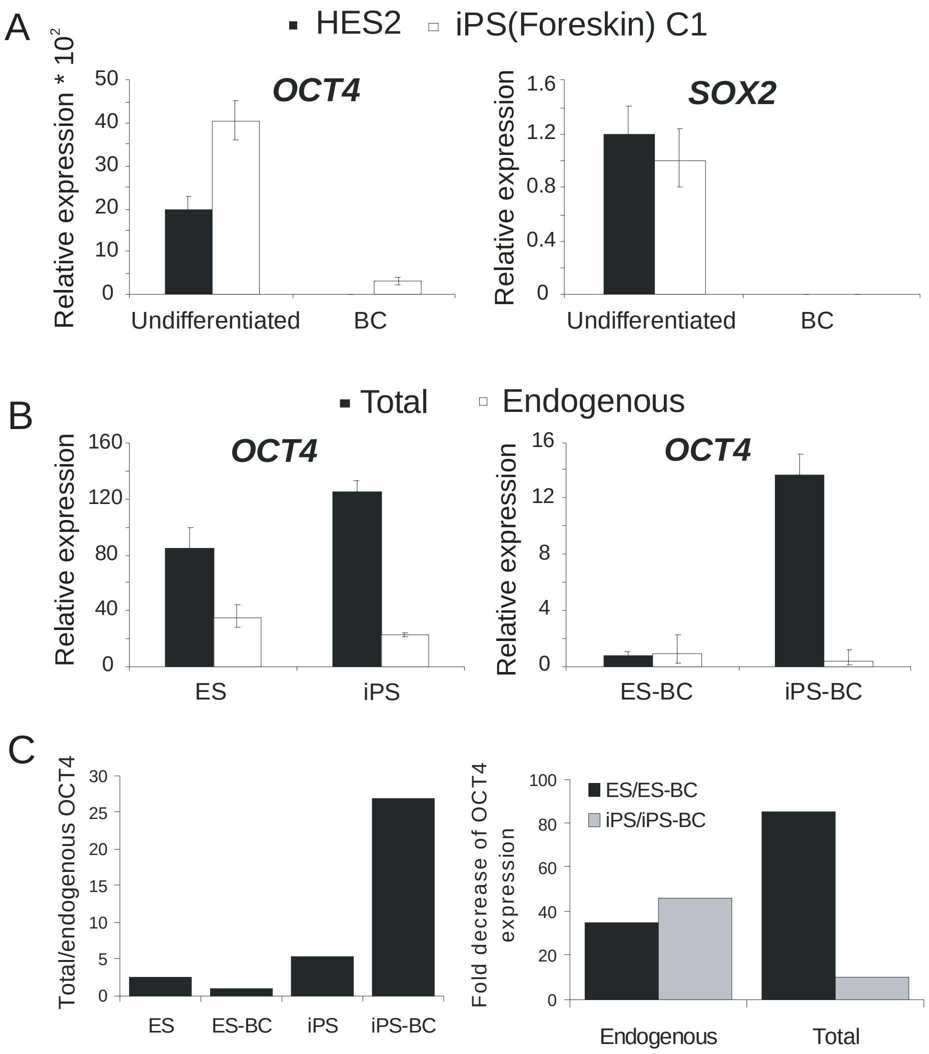

Figure S3 - Expression of pluripotency genes in beating clusters (BC) derived from human iPS and ES cells. Quantitative RT-PCR analyses were performed with RNA samples isolated from undifferentiated iPS and ES cells and their corresponding BCs microdissected at day 18 of differentiation. **A.** Expression of total OCT4 and total SOX2; **B.** Relative expression of total and endogenous OCT4 in undifferentiated ES and iPS cells (left panel) and BCs (right panel). **C.** Ratios between the total and endogenous OCT4 levels (left panel) and fold decrease of endogenous and total OCT4 expression in BCs as compared to undifferentiated cells (right panel). The data in panels A and B are means of triplicate analyses ± SEM.

Table S1 - Sequences and amplification conditions of primer pairs used for RT-PCR analyses of total RNA isolated from undifferentiated and differentiated human iPS and ES cells.

Gene	NCBI Accession	Sequence (5' to 3')	Amplicon size (nt)	Annealing temp., °C
OCT3/4	NM_002701	**F:** AGGGCAAGCGATCAAGCA<br>**R:** GGAAAGGGACCGAGGAGTA	168	60
NANOG	NM_024865.2	**F:** ACTAACATGAGTGTGGATCC<br>**R:** TCATCTTCACACGTCTTCAG	930	60
SOX2	NM_003106.2	**F:** ATGCACCGCTACGACGTGA<br>**R:** CTTTTGCACCCCTCCCATTT	437	60
NKx2.5	NM_004387.2	**F:** CCTCAACAGCTCCCTGACTC<br>**R:** GGGGACAGCTAAGACACCAG	201	60
MLC2v	NM_000432.3	**F:** ACAGGGATGGCTTCATTGAC<br>**R:** CCTCCTCCTTGGAAAACCTC	288	60
Troponin T	X79859.1	**F:** ATCCCCGATGGAGAGAGAGT<br>**R:** CCTCCTGTTCTCCTCCTCCT	264	60
α-Actinin 2	NM_001103.1	**F:** GGCACCCAGATTGAGAACAT<br>**R:** CCTGAATAGCAAAGCGAAGG	268	60
OCT3/4 (total)	NM_002701	**F:** CAGTGCCCGAAACCCACAC<br>**R**: GGAGACCCAGCAGCCTCAAA	161	60
OCT3/4 (endogenous)	NM_002701	**F:** AGTTTGTGCCAGGGTTTTTG<br>**R:** ACTTCACCTTCCCTCCAACC	113	60
CKLF	NM_001040138	**F:** GCAGCCAGCTGAGAAGAGTT<br>**R:** ATCTTCACGTGGCCTTTCAC	122	60

COMT	NM_007310	**F:** ACATGGTCTTCCTCGACCAC<br>**R:** TCACGTTGTCAGCCAGTAGC	108	60
DYNLT3	NM_006520	**F:** TGGGGTTTTAGGTGGTGAAG<br>**R:** CTCTTCTGGACCACTGCACA	141	60
MGC3207	NM_0010317<br>27	**F:** ATGGACCCCAGATGTAACCA<br>**R:** AGCTCCCTGCACTAGGAACA	131	60
MGMT	NM_002412	**F:** CCGTTTGCGACTTGGTACTT<br>**R:** CTCACAACCAGACAGCTCCA	101	60
NME4	NM_005009	**F:** TGTGGTTCCAGAGCAGTGAG<br>**R:** GGTTCTTGCTGACGGAGGTA	135	60
OXCT1	NM_000436	**F:** TGGGACCCTGGTACAAGAAG<br>**R:** CCCTGTAATTGCTTCCTCCA	133	60
PTGR1	NM_012212	**F:** CAGTTCCTTGGAGAGCTTGG<br>**R:** AAGTGCTTCTTCAGGGTCCA	119	60
ZNF167	NM_018651	**F:** GGAGCACTGCTTTCCAGAAG<br>**R:** AGATTTCGCAGACAGGAGGA	117	60
ZNF626	NM_145297	**F:** TCAGAGCTCCAGGTCTGGTT<br>**R:** AGGAGGTCCCGGTGTCTTAG	100	60

Table S2 - Field potential (FP) frequencies of microdissected beating clusters derived from differentiated human iPS and ES cells at day 18-20 of differentiation.

FP frequencies were measured by MEA in the absence or presence of pharmacological stimulation of beta-adrenergic (isoproterenol, ISO, 1 µM) or muscarinic (Carbachol, CCh, 10 µM) receptors. Wo – washout.

hES-CM (ISO), Hz				hiPS-CM (ISO), Hz			
**Exp. No.**	**Control**	**ISO**	**Wo**	**Exp. No.**	**Control**	**ISO**	**Wo**
1	1.56	2.71	2.38	1	0.84	1.43	0.97
2	0.65	1.65	1.47	2	0.56	1.17	0.83
3	1.04	2.19	1.13	3	0.76	1.26	1.01
4	0.51	1.28	0.65	4	1.12	2.14	1.64
				5	1.48	2.74	1.74
**Mean**	0.94	1.96	1.41	**Mean**	0.95	1.75	1.24
**SEM**	0.47	0.63	0.73	**SEM**	0.23	0.44	0.36
**p-value**	└ 0.04 ┘			**p-value**	└ 0.04 ┘		
**hES-CM (CCh), Hz**				**hiPS-CM (CCh), Hz**			
**Exp. No.**	**Control**	**CCh**	**Wo**	**Exp. No.**	**Control**	**CCh**	**Wo**
1	2.61	0.25	1.28	1	0.87	0.57	0.62
2	0.62	0.2	0.48	2	1.68	0	0.43
3	1.46	1.07	0.95	3	0.78	0.32	0.24
4	1.08	0	0.48	4	1.06	0	0.36
5	0.84	0.24	0.46	5	1.59	0.26	0.88
				6	0.66	0.22	0.33
**Mean**	1.32	0.35	0.73	**Mean**	1.11	0.23	0.48
**SEM**	0.78	0.42	0.37	**SEM**	0.43	0.21	0.24
**p-value**	└ 0.04 ┘			**p-value**	└ 0.01 ┘		

Table S3 - Analysis of spontaneous Ca^{2+} transients (calcium release curves) of iPS-BCs and hES-BCs as detected under perfusion with Tyrodes's solution. No statistically significant difference (p>0.05) was found comparing each parameter of the two different cell lines. For comparison, data generated from H9.2 hES cell line [12] and stimulated right auricular muscle strip preparations from non-failing human myocardium [16] are shown.

	Basal [F340/F380]	Maximum [F340/F380]	Amplitude [F340/F380]	Average frequency [Hz]	Transient time [s]	Upstroke velocity, V_{max} [F340/F380/s]	Decay velocity, V_{min} [F340/F380/s]	Half decay time [s]	Time to peak [s]	No. of BCs [n]	Transients analyzed [n]
iPS-BCs, this study	0.501±0.020	0.613±0.015	0.112±0.026	0.517±0.072	0.874±0.093	1.462±0.309	-0.508±0.096	0.182±0.016	0.261±0.042	7	98
hES-BCs, this study	0.526±0.018	0.634±0.021	0.108±0.015	0.572±0.104	0.853±0.065	1.561±0.213	-0.495±0.069	0.207±0.014	0.178±0.020	7	109
H9.2 BCs, Kehat 2001				0.900±0.110	0.465±0.180			0.143±0.094	0.130±0.027	7	
Human heart, Brixius 1997				1,000 (stimulated)	0.539±0.031			0.177±0.009	0.053±0.003	24	

Table S4 - Simultaneous observation of contractions and Ca^{2+} transients reveals an intact cardiac excitation-contraction coupling. Each Ca^{2+} transient is accompanied by a single contraction of the examined beating cluster.

Beating cluster	Contractions detected<br>t = 30 s	Transients recorded<br>t = 30 s
hiPS-BC 1	11	11
hiPS-BC 2	16	16
hiPS-BC 3	6	6
hiPS-BC 4	15	15
hiPS-BC 5	16	16
hiPS-BC 6	21	21
hiPS-BC 7	23	23
hES-BC 1	10	10
hES-BC 2	22	22
hES-BC 3	12	12
hES-BC 4	17	17
hES-BC 5	33	33
hES-BC 6	11	11
hES-BC 7	15	15

Table S5 - List of samples used for global gene expression profiling using Illumina platform.

No.	Sample name	Description	Cell line	Passage number	Origin of a cell line
**1**	hESC_undiff _replicate 1	human ES cells (hESC), undifferentiated	HES-2	129	ES Cell International, Singapore
**2**	hESC_undiff _replicate 2			132	
**3**	hESC_undiff _replicate 3			97	
**4**	hESC_CM _replicate 1	hESC-derived contracting areas, day 18 of in vitro differentiation		129	
**5**	hESC_CM _replicate 2			132	
**6**	hESC_CM _replicate 3			97	
**7**	hiPSC_undiff _replicate 1	human induced pluripotent stem cells (hiPSC), undifferentiated	iPS(Foreskin), clone 1	20	James Thomson, University of Wisconsin
**8**	hiPSC_undiff _replicate 2			23	
**9**	hiPSC_undiff _replicate 3			28	
**10**	hiPSC_CM _replicate 1	hiPSC-derived contracting areas, day 18 of in vitro differentiation		20	
**11**	hiPSC_CM _replicate 2			23	
**12**	hiPSC_CM _replicate 3			28	

**13**	fetal heart _replicate 1a	Normal fetal heart, pooled from 34 fetuses	human tissue	n/a	Clontech (Cat. No. 636583, LOT 8101346A)
**14**	fetal heart _replicate 1b				
**15**	fetal heart _replicate 1c				
**16**	adult heart _replicate 1a	Normal adult heart, pooled from 3 individuals	human tissue	n/a	Clontech (Cat. No. 636532, LOT 7120603A)
**17**	adult heart _replicate 1b				
**18**	adult heart _replicate 1c				

Supplementary results

Expression of endogenous and transgenic pluripotency genes

Since the reactivation of lentiviral pluripotency genes in differentiated iPS cell-derivatives may affect their transcriptome, we have determined the expression of ES cell-specific transcripts *OCT4* and *SOX2* in undifferentiated iPS and ES cells and in microdissected BCs derived from them at day 18 of differentiation. The expression of *OCT4* and *SOX2* was high in undifferentiated iPS and ES cells and dropped significantly in BCs (Additional file 1, Figure S3A). While *SOX2* gene expression was apparently abolished in iPS-BCs and ES-BCs, expression of *OCT4*, which was approximately 2-fold higher in iPS cells compared to ES cells, was still detectable in BCs derived from iPS cells and to a much lesser extent in ES-BCs (Additional file 1, Figure S3A).

Since the iPS cells used in this study contain stably integrated lentiviral expression cassettes, the BCs were assayed for residual expression of a viral transgene, which may occur due to its incomplete silencing or reactivation. We have focused on the *OCT4* gene. In order to distinguish between endogenous and viral *OCT4* transcripts, we used primers located in the coding region to determine the total and primers in the 5’ untranslated region to determine the endogenous *OCT4* gene expression (Additional file 1, Table S1). The expression of the virally encoded *OCT4* gene can then be estimated from the increase of ratio between total and endogenous gene expression above the value found in undifferentiated ES cells. In our analyses, the ratio between total and endogenous *OCT4* gene expression in ES cells was 2.4 (Additional file 1, Figure S3B), and this value was used as a reference for comparison with ratios found in iPS cells and iPS-BCs. Both undifferentiated iPS cells and iPS-

BCs expressed much higher levels of total *OCT4* gene than the endogenous one (Additional file 1, Figure S3B**)** giving rise to total/endogenous ratio of, respectively, 5.4 and 27 (Additional file 1, Figure S3C, left panel). These data suggest that the viral *OCT4* transgene is actively transcribed in both undifferentiated iPS cells and iPS-BCs, and that the major fraction of *OCT4* transcripts in beating areas isolated from iPS cells is of a lentiviral origin. This conclusion is in agreement with the finding that the total *OCT4* gene expression was downregulated to a much lesser extent in iPS-BCs (10-fold) than in ES-BCs (85-fold) compared to their respective undifferentiated counterparts (Additional file 1, Figure S3C, right panel). In contrast, the degree of downregulation of the endogenous *OCT4* gene expression was similar for iPS (46-fold) and ES cells (35-fold) (Additional file 1, Figure S3C, right panel). Thus, although *OCT4* expression significantly declined in the course of differentiation of both iPS-BCs and ES-BCs, the downregulation in iPS-BCs was much lesser due to incomplete silencing of the lentiviral transgene.

Since BCs are composed of multiple cell types, they still may harbor small amounts of pluripotent cells that did not differentiate. Although the estimation of silencing or reactivation of viral transgenes may be less accurate in this case, it is still possible to argue, based on expression data obtained with primers specific for endogenous and total transcripts, that incomplete silencing of lentiviral transgenes indeed occurs. As assessed with qRT-PCR for endogenous *OCT4* transcripts in Additional file 1, Figure S3C (in this assay virally encoded *OCT4* can not be detected), the degree of reduction of *OCT4* expression in the course of differentiation to BCs was very similar in ES and iPS cells, suggesting that iPS-BCs and ES-BCs contain comparable amounts of cells expressing OCT4 (e.g. pluripotent stem cells and/or germ line stem cells). In contrast, total *OCT4* mRNA levels comprising both the endogenous and viral transcripts

decreased to a much lower extent in iPS-BCs than in ES-BCs. Therefore, discrepancy between total and endogenous OCT4 mRNA levels in iPS-BC and ES-BCs is strongly indicative of incomplete silencing of the lentiviral transgene in iPS-BCs. This conclusion is supported by our observation that murine *Oct4* mRNA expressed from a retroviral vector was detected in highly purified murine iPS-CMs that were generated by drug selection using our transgenic iPS cell line (*Fatima A., manuscript in preparation*). Since these iPS-CMs were not contaminated by pluripotent stem cells, the *Oct4* transgene in these cells can only be expressed in CMs. This observation clearly supports the conclusion that viral pluripotency factors may still be ectopically expressed due to incomplete silencing even in highly purified mature iPS cell-derivatives. Despite this incomplete silencing of *Oct4* transgene, we could not detect any functional abnormalities in iPS-CMs. Hochedlinger and coworkers (Cell 121:465-477, 2005) showed that ectopic Oct4 expression in the heart of adult mice did not lead to any detectable alterations of cell phenotype. In contrast, they found that the activation of Oct4 in epithelial progenitor and/or stem cells results in dysplastic growth in epithelial tissues in intestine and skin. However, Oct4 expression in already differentiated cells of the intestine or hair follicle had no effect on the cellular phenotype. Therefore, ectopic Oct4 expression may have only limited detrimental effects in specific cell types at early developmental stage and would not affect the function of many other cell types.

Functional characterization of iPS-CMs

A few recent studies have demonstrated that human iPS and ES cells possess comparable cardiogenic potential and that iPS-CMs have similar structural and electrophysiological properties [1-5]. In the present study, we corroborate these

observations and show that early stage CMs generated from human iPS cells (Foreskin C1) by differentiation in co-culture with END2 feeder cells exhibit a high resemblance to early stage CMs derived from conventional human ES cells (HES-2). The onset of spontaneous beating, the yield of beating clusters and their beating frequencies (Additional file 1, Figure S2), expression of selected cardiac genes and structural organization of sarcomeric proteins (Figure 1) were all comparable between iPS and ES cells. In addition, MEA analyses revealed that the response of human iPS-CMs to pharmacological stimulation of β-adrenergic and muscarinic signaling pathways was similar to those of human ES-CMs (Figure 2 and Additional file 1, Table S2). Furthermore, we demonstrate that spontaneous Ca^{2+}-release and caffeine-induced Ca^{2+}-transients in human iPS-CMs are also indistinguishable from those observed in ES-CMs (Figure 3 and Additional file 1, Tables S3 and S4). Strong caffeine-induced Ca^{2+}-transient indicates the presence of well-developed ryanodine-gated intracellular Ca^{2+} stores in the SR, which is the hallmark of the cardiac phenotype and has been demonstrated for human ES-CMs in previous studies [6,7], and recently described for human iPS-CMs [8]. The observed effect resembles the effect of caffeine on the spontaneous Ca^{2+} release in native rat ventricular myocytes [9], guinea-pig atrial trabeculae [10] and sheep cardiac Purkinje fibers [11]. It is noteworthy that all of the aggregates in this study showed the increased Ca^{2+} levels in a transient response to caffeine. During ongoing caffeine perfusion (1 min) we observed a cessation of spontaneous transients as shown in Figure 3B in about half of the experiments. This was most likely due to differences in the concentration of the drug that can be obtained in the cells of a particular aggregate or in the cellular composition of microdissected BCs. After washout, all aggregates recovered from the caffeine treatment indicating the effect to be reversible (data not shown). In agreement with other studies, the Ca^{2+}-

transients in our study indicate a fast initial rise in systolic $[Ca^{2+}]_i$ and a slower decay [6,12]. However, the averaged time to peak systolic $[Ca^{2+}]_i$ and the half-peak relaxation values are a bit slower than in the study of Kehat and co-workers (see Additional file 1, Table S3) [12]. This difference may be due to a slower average beating frequency in our experiments and to different experimental conditions (e.g. Tyrode's solution). It is also possible, that the EBs we produce with the END-2 differentiation protocol contain more ventricular cells (see ref. [13]) and therefore beat with a lower frequency compared to the H9.2-BCs that were generated in a suspension differentiation protocol based on EB formation [12]. Liu and colleagues did not publish detailed values [6]. All their data is presented in form of graphs, making a detailed comparison difficult. In addition, they did not measure whole EBs but isolated cells. In several other publications Ca^{2+} transients of human ES cell derived CMs were investigated. However, detailed parameters of these transients were not shown [6,7,14,15]. Comparison of our data with the published data from "*in vivo scenario*" revealed that Ca^{2+} transients obtained in our study for iPS- and ES-CMs are comparable to those in stimulated right auricular muscle strip preparations from non-failing human myocardium, except the fact that the time to peak value was much faster *in vivo* [16] (Additional file 1, Table S3). This difference may be explained by the different types of CMs present in an EB, while the heart probe that was used in reference [16] consisted of atrial cells. We have further carefully microscopically examined spontaneous contractions and Ca^{2+} transients in order to determine whether each transient is accompanied by a contraction and vice versa. (Additional file 1, Table S4). By looking at the first 30 seconds of each measurement we observed that every Ca^{2+} transient comes along with a single contraction. This synchrony of resulting

contraction is a manifestation of an intact cardiac excitation-contraction coupling and has been reported earlier for H9.2 derived cardiomyocytes [12].

Microarray analysis of human ES cell specific and enriched genes

ES cell-specific transcripts shown in Additional file 6 (Tables S12 and S13) were expressed in average at approximately 5-6-fold higher levels (ranging from 2-30 fold) in ES and iPS cells compared to the corresponding microdissected beating clusters (BCs), demonstrating their ES cell-specificity. As expected, the expression of ES cell-specific genes in fetal hearts (FH) and adult hearts (AH) was absent or, in few cases (*AEN, SLC5A6, ORC2L and GPR23/LPAR4*), very low (Additional file 6, Table S12). In addition, ratios of mean expression values between hES and hiPS, hES-BC and hiPS-BC, and FH and AH were for most genes close to 1 indicating highly similar expression profiles of pluripotency genes between these samples. However, one gene was expressed at significantly higher levels in ES cells compared to iPS cells (*CYP26A1*, 3.8-fold) and two genes were expressed at significantly higher levels in iPS cells compared to ES cells (*ABHD9*, 5.5-fold; *NANOG*, 6.1-fold), ($p<0.05$, Additional file 6, Table S12). Consensus genes that have been reported to be enriched in human ES cells were expressed at approximately 5-fold higher levels (ranging from 1.8- to 73-fold for *PODXL* and *ZSCAN10*, respectively) in ES and iPS cells compared to the corresponding microdissected beating clusters (Additional file 6, Table S13). Ratios of mean expression values between human ES and iPS cells as well as between hES-BC and hiPS-BC were for most ES cell enriched genes also close to 1 indicating highly similar expression profiles of these genes between these cell types.

Although the expression of ES cell-enriched genes in beating clusters, FH and AH was in most cases absent or very low, some transcripts were also elevated in iPS-BC

and ES-BC and/or AH and FH (*SH3GL2, GPC4, CEBPZ, USP9X, ALPL, SEMA6A, SEPHS1, SALL2, UNG, SNRPN, MGST1, PSIP1, CRMP1, UGP2, NASP, ITGB1BP3*) (Additional file 6, Table S13). Five transcripts from this list were expressed at higher levels in FH compared to AH (*CEBPZ, SEMA6A, PSIP1, CRMP1, NASP*), while few others were detected at higher levels in AH compared to FH (*SNRPN, MGST1, ITGB1BP3*). Two genes in this table were expressed at significantly higher levels in ES cells compared to iPS cells (*ZFP42*, 14.6-fold; *USP9X*, 2.1-fold), ($p<0.05$, Additional file 6, Table S13).

Microarray analysis of cardiac enriched genes

The mean of expression values of all selected genes in Additional data file 6 (Table S14**)** for each cell type demonstrates the cardiac specificity of the genes, which exhibit in average about 30-fold higher expression in ES and iPS cell-derived BCs and more than 50-fold higher expression in FH and AH compared to expression in undifferentiated ES and iPS cells. Among the most upregulated genes in iPS-BCs and ES-BCs were known cardiac genes that encode for structural proteins (*MYH6, MYL7, MYL4, MYOM1, MYBPC3, TNNT2, TNNC1, ACTC1, ACTN2, TMOD1*), hormones and cytokines (*NPPA, BMP2, BMP5, DKK1*), transcription regulators (*SMYD1, SMARCD3, ISL1, NKX2.5, TBX2, GATA4, GATA5, MEF2C, HOPX, FOXC1, MSX1*) and ion channels and their modulators (*ATP2A2, RYR2, PLN, HRC*) (Additional file 8, Tables S16-S19). Among the most downregulated genes in both types of BCs were pluripotency markers *ZSCAN10, DPPA4, DNMT3B,, OCT4, GAL, ZIC3, SOX2, LECT1, LIN28, EPHA1, PIM2, CYP26A1, SALL4* and others (Additional file 8, Tables S16-S19). Thus, iPS-BCs contain CMs that are highly similar to their respective ES cell counterparts.

Despite some exceptions, average ratios (hiPS/hES, hiPS-BC/hES-BC, FH/AH) for most selected genes have a value close to 1 confirming highly similar expression profiles between these sample pairs. Several genes previously reported to be enriched in human ES cell-derived beating clusters [17,18] were also found to be highly expressed in hES-BCs and hiPS-BC but were absent in both FH and AH indicating that they are not cardiospecific (e.g. *AFP, TF, FGB, TTR, FGG, GABRP, SERPINA1, AHSG*) or are expressed only transiently in early cardiac myocytes or their progenitors (e.g. *ISL1, GATA5*), which may be present in BCs but are absent from FH and AH (Additional file 6, Table S14). The expression of liver-specific genes *AFP, TF, FGG, TTR, SERPINA1* and *AHSG* suggests the presence of endodermal derivatives in both ES-BCs and iPS-BCs, which is in agreement with the findings of others [17]. Other genes known to be involved in cardiogenesis such as *TBX1, CLDN5, MEOX1, MYOCD, GATA6,* and *FOXH1* were not expressed. Despite very high fold change in comparisons of mean expression values between undifferentiated cells and their corresponding BCs some cardiac enriched genes shown in Additional file 6 (Table S14**)** were not scored as differentially expressed with statistical significance. This was due to variability in expression levels between replicates in individual experimental groups (cardiac specific genes *MYH7B, MYL3* and *MYOZ2*, and liver-specific genes *TF, FGG, TTR*, and *AFP*).

Supplementary methods

Flow cytometry

For flow cytometry cells were dissociated with 0.05% Trypsin/0.2 g/L EDTA, cell clumps were removed by passing through the cell strainer (70 µm pore size, BD Falcon®). Single cell suspensions were incubated with primary antibodies against SSEA-4 (clone 813-70) or Tra-1-81 (clone 1-80) or corresponding isotype control antibodies followed by phycoerythrin (PE)-conjugated secondary antibodies (Santa Cruz CA, USA, www.scbt.com). Data were collected with a FACScan and analyzed by CellQuest Pro software (BD Pharmingen, San Diego, CA, USA, http://www.bdbiosciences.com). Propidium iodide (PI) staining was performed in order to distinguish dead cells. At least 10,000 live (PI negative) events were acquired for each sample.

Alkaline phosphatase staining

Undifferentiated ES and iPS cell cultures were fixed with 100% methanol, air dried and incubated for 15 minutes at 37°C with staining solution containing 200 µg/ml Napthol AS-MX and 0.9 mg/ml Fast Red TR salt™ in 0.1 M Tris-HCl, pH 9.2. Both reagents were obtained from Sigma-Aldrich, St.Louis, MO, USA, www.sigma-aldrich.com). Stainings were terminated by rinsing with PBS and analysed by light microscopy.

qRT-PCR validation of microarray data

Microarray results obtained for undifferentiated iPS and ES cells were validated using Human Embryonic Stem Cell 96 StellARray™ qPCR array (Lonza, Cologne, Germany, www.lonza.com). Each StellARray plate contains validated primer pairs for

amplification of 57 different pluripotency and 37 different differentiation genes in addition to one housekeeping gene and one genomic DNA control. The same RNA samples that were prepared for microarray analyses were DNase I-treated and 500 ng of RNA was reverse transcribed as described in the Methods section. The cDNA probes were diluted 1:10 and added (equivalent to 1 μl/well) to the SYBR Advantage qPCR Premix (Takara Bio Europe/Clontech, Saint-Germain-en-Laye, France, www.clontech-europe.com) prepared with ROX dye and sterile water according to the manufacturers instructions. 20 μl of this supermix were distributed into each well of a 96 well StellARay plate. Plate was then firmly sealed and centrifuged to ensure that all reagents were at the bottom of wells. StellARay plate was soaked for 15 minutes at room temperature to allow for dissolution of the lyophilized primers and the plate was loaded into a preprogrammed 7500 Fast System Real Time Cycler (Applied Biosystems). For each group the analysis was performed in triplicates and data were analyzed using the Global Pattern Recognition™ Software (Lonza).

Supplementary references

1. Tanaka T, Tohyama S, Murata M, Nomura F, Kaneko T, Chen H, Hattori F, Egashira T, Seki T, Ohno Y *et al*: **In vitro pharmacologic testing using human induced pluripotent stem cell-derived cardiomyocytes**. *Biochem Biophys Res Commun* 2009, **385**(4):497-502.

2. Yokoo N, Baba S, Kaichi S, Niwa A, Mima T, Doi H, Yamanaka S, Nakahata T, Heike T: **The effects of cardioactive drugs on cardiomyocytes derived from human induced pluripotent stem cells**. *Biochem Biophys Res Commun* 2009, **387**(3):482-488.

3. Zhang J, Wilson GF, Soerens AG, Koonce CH, Yu J, Palecek SP, Thomson JA, Kamp TJ: **Functional cardiomyocytes derived from human induced pluripotent stem cells**. *Circ Res* 2009, **104**(4):e30-41.

4. Gai H, Leung EL, Costantino PD, Aguila JR, Nguyen DM, Fink LM, Ward DC, Ma Y: **Generation and characterization of functional cardiomyocytes using induced pluripotent stem cells derived from human fibroblasts**. *Cell Biol Int* 2009, **33**(11):1184-1193.

5. Zwi L, Caspi O, Arbel G, Huber I, Gepstein A, Park IH, Gepstein L: **Cardiomyocyte differentiation of human induced pluripotent stem cells**. *Circulation* 2009, **120**(15):1513-1523.

6. Liu J, Fu JD, Siu CW, Li RA. **Functional sarcoplasmic reticulum for calcium handling of human embryonic stem cell-derived cardiomyocytes: insights for driven maturation.** *Stem Cells.* 2007;**25**:3038-3044.

7. Satin J, Itzhaki I, Rapoport S, Schroder EA, Izu L, Arbel G, Beyar R, Balke CW, Schiller J, Gepstein L. **Calcium handling in human embryonic stem cell-derived cardiomyocytes.** *Stem Cells.* 2008;**26**:1961-1972.

8. Germanguz I, Sedan O, Zeevi-Levin N, Shtreichman R, Barak E, Ziskind A, Eliyahu S, Meiry G, Amit M, Itskovitz-Eldor J *et al*: **Molecular characterization and functional properties of cardiomyocytes derived from human inducible pluripotent stem cells**. *J Cell Mol Med* 2009.

9. Trafford AW, Sibbring GC, Diaz ME, Eisner DA: **The effects of low concentrations of caffeine on spontaneous Ca release in isolated rat ventricular myocytes**. *Cell Calcium* 2000, **28**(4):269-276.

10. Glitsch HG, Pott L: **Spontaneous tension oscillations in guinea-pig atrial trabeculae**. *Pflugers Arch* 1975, **358**(1):11-25.

11. Nieman CJ, Eisner DA: **Effects of caffeine, tetracaine, and ryanodine on calcium-dependent oscillations in sheep cardiac Purkinje fibers**. *J Gen Physiol* 1985, **86**(6):877-889.

12. Kehat I, Kenyagin-Karsenti D, Snir M, Segev H, Amit M, Gepstein A, Livne E, Binah O, Itskovitz-Eldor J, Gepstein L. **Human embryonic stem cells can differentiate into myocytes with structural and functional properties of cardiomyocytes.** *J Clin Invest.* 2001;**108**:407-414.

13. Mummery C, Ward-van Oostwaard D, Doevendans P, Spijker R, van den Brink S, Hassink R, van der Heyden M, Opthof T, Pera M, de la Riviere AB, Passier R, Tertoolen L. **Differentiation of human embryonic stem cells to cardiomyocytes: role of coculture with visceral endoderm-like cells**. *Circulation.* 2003;**107**:2733-2740

14. Cao F, Wagner RA, Wilson KD, Xie X, Fu JD, Drukker M, Lee A, Li RA, Gambhir SS, Weissman IL, Robbins RC, Wu JC. **Transcriptional and functional profiling of human embryonic stem cell-derived cardiomyocytes**. *PLoS ONE.* 2008;**3**:e3474.

15. Stevens KR, Pabon L, Muskheli V, Murry CE. **Scaffold-free human cardiac tissue patch created from embryonic stem cells.** *Tissue Eng Part A.* 2009;**15**:1211-1222.

16. Brixius K, Pietsch M, Hoischen S, Muller-Ehmsen J, Schwinger RH. **Effect of inotropic interventions on contraction and Ca2+ transients in the human heart.** *J Appl Physiol.* 1997;**83**:652-660.

17. Synnergren J, Akesson K, Dahlenborg K, et al. **Molecular signature of cardiomyocyte clusters derived from human embryonic stem cells.** *Stem Cells* 2008;**26**:1831-1840.

18. Beqqali A, Kloots J, Ward-van Oostwaard D, Mummery C, Passier R. **Genome-wide transcriptional profiling of human embryonic stem cells differentiating to cardiomyocytes**. *Stem Cells* 2006;**24**:1956-1967.
