# Supplementary material for: Global transcriptional profiles of beating clusters derived from human induced pluripotent stem cells and embryonic stem cells are highly similar
Source: BMC Dev Biol. 2010 Sep 15;10:98. doi: 10.1186/1471-213X-10-98 (PMC2946283; doi:10.1186/1471-213X-10-98)
Supplement: Additional file 5 — Expression levels of selected genes included in GO-terms that were found to be overrepresented in iPS-BCs compared to ES-BCs. This PDF file contains the Table S11 displaying the expression levels of selected fibroblastassociated genes found to be overexpressed in iPS-BCs compared to ES-BCs. [file 1471-213X-10-98-S5.PDF]

Table S11 – Expression levels of selected genes included in GO-terms that were found to be overrepresented in iPS-BCs compared to ES-BCs.

Gene symbol[1]	Mean ES	Mean iPS	Fold change[2] iPS/ ES	p-value	Mean ES-BC	Mean iPS-BC	Fold change[2] iPS-BC/ ES-BC	p-value
LAMB1	318	209	-1,5	>0.05	295	678	2,3	0.018
DCN	87	95	1,1	>0.05	4222	15098	3,6	0
LUM	84	90	1,1	>0.05	1050	12152	11,6	0
COL1A1	588	412	-1,4	>0.05	3575	9188	2,6	0.036
COL1A2	659	496	-1,3	>0.05	3350	8542	2,6	0,0035
COL3A1	87	89	1,0	>0.05	8823	24651	2,8	1,0E-4
COL4A1	2738	2105	-1,3	>0.05	3256	7624	2,3	0,019
COL5A1	298	328	1,1	>0.05	1481	5856	4,0	0,021
COL5A2	879	1073	1,2	>0.05	2899	9739	3,4	0
COL6A3	112	97	-1,2	>0.05	998	4151	4,2	0
COL12A1	177	134	-1,3	>0.05	103	351	3,4	5,0E-4
CD36	84	90	1,1	>0.05	112	277	2,5	0,0024
CD44	252	193	-1,3	>0.05	424	1190	2,8	0,0059
CD47	149	198	1,3	>0.05	393	820	2,1	0,0014
CTGF	2649	3036	1,2	>0,05	4333	9096	2,1	0,0019
HOXA5	121	107	0,9	>0,05	111	260	2,3	0
HOXB5	87	93	1,1	>0,05	343	873	2,5	6,0E-4
NR2F2	97	106	1,1	>0,05	116	295	2,5	0
SNAI2	81	83	1,0	>0,05	391	1328	3,4	0
Mean±SD			1,0±0,2	Mean±SD			3,3±2,1	

[1] Abbreviations: LAMB1 – laminin beta 1; DCN – decorin; LUM – lumican; COL*x*A*y* - collagen, type *x*, alpha *y*, CTGF – connective tissue growth factor; HOXA5 and HOXB5 – homeobox protein A5 and B5; NR2F2 - COUP transcription factor 2; SNAI2 – zinc finger transcription factor of the Snail family.

[2] The fold-change was calculated by dividing the mean intensity of the genes in one group by that in the other group as indicated above each column. If this number was less than one, the negative reciprocal was used.
