# Supplementary material for: Global transcriptional profiles of beating clusters derived from human induced pluripotent stem cells and embryonic stem cells are highly similar
Source: BMC Dev Biol. 2010 Sep 15;10:98. doi: 10.1186/1471-213X-10-98 (PMC2946283; doi:10.1186/1471-213X-10-98)
Supplement: Additional file 6 — Expression levels of selected human ES cell- and cardiacspecific or enriched genes across all samples. This is a PDF file in a landscape format containing Table S12 (list of 27 ES cell-specific genes and their expression levels across all samples), Table S13 (list of 49 human ES cell-enriched genes with their expression levels in all samples) and Table S14 (list of 83 cardiac specific or enriched genes and their expression levels across all samples). [file 1471-213X-10-98-S6.PDF]

Table S12 - Expression of 27 human ES cell-specific genes in undifferentiated human ES and iPS cells, microdissected spontaneously beating clusters derived from human ES (ES-BC) and iPS cells (iPS-BC) as well as human fetal heart (FH) and adult heart (AH)[1]

Probeset	Access. #	Symbol	Genename	Mean iPS	Mean ES	Ratio iPS/ ES	Mean iPS-BC	Mean ES-BC	Ratio iPS-BC/ ES-BC	Mean FH	Mean AH	Ratio FH/ AH
1340551	NM_024674	LIN28	lin-28 homolog (C. elegans)	9062	9090	1,00	695	693	1,00	75	71	1,05
5270072	NM_057157	CYP26A1	cytochrome P450, family 26, subfamily A, polypeptide 1	2332	8854	0,26	250	300	0,83	91	93	0,98
670414	NM_003413	ZIC3	Zic family member 3 (odd-paired homolog, Drosophila)	1896	3419	0,55	92	354	0,26	75	75	1,01
5490358	NM_019079	L1TD1 (FLJ10884)	LINE-1 type transposase domain containing 1, ECAT11	2945	2817	1,05	157	106	1,49	84	76	1,12
4480288	NM_022767	AEN (FLJ12484)	apoptosis enhancing nuclease	2201	2339	0,94	1171	1196	0,98	210	411	0,51
6370553	NM_005232	EPHA1	EPH receptor A1	2012	1616	1,25	174	125	1,39	100	92	1,09
770615	NM_002701	POU5F1	POU class 5 homeobox 1	1086	1537	0,71	74	83	0,89	75	74	1,02
5050040	NM_021095	SLC5A6	solute carrier family 5 (sodium-dependent vitamin transporter),	1824	1331	1,37	873	687	1,27	335	503	0,67

			member 6									
2480341	NM_001789	CDC25A	cell division cycle 25 homolog A (S. pombe)	1318	1103	1,19	397	303	1,31	141	96	1,47
6620475	NM_006190	ORC2L	origin recognition complex, subunit 2-like (yeast)	1147	1069	1,07	363	401	0,91	273	294	0,93
7570181	NM_018518	MCM10	minichromosome maintenance complex component 10	1185	1039	1,14	546	377	1,45	176	95	1,86
7040161	NM_018063	HELLS	helicase, lymphoid-specific	660	839	0,79	204	150	1,36	103	77	1,33
6200072	NM_006143	GPR19	G protein-coupled receptor 19	577	683	0,84	259	283	0,91	107	84	1,27
5260685	NM_007194	CHEK2	CHK2 checkpoint homolog (S. pombe)	708	670	1,06	180	224	0,80	121	104	1,16
7550242	NM_003865	HESX1	HESX homeobox 1	258	595	0,43	131	112	1,17	92	75	1,22
6660333	NM_004153	ORC1L	origin recognition complex, subunit 1-like (yeast)	440	445	0,99	132	117	1,13	86	93	0,92
70209	NM_001986	ETV4	ets variant 4	529	404	1,31	127	131	0,97	98	95	1,03
770634	NM_024504	PRDM14	PR domain containing 14	342	398	0,86	83	94	0,88	93	83	1,11
1450274	NM_021195	CLDN6	claudin 6	609	366	1,66	156	133	1,17	84	83	1,01
5050181	NM_017669	FLJ20105,	excision repair cross-	341	354	0,96	133	104	1,28	82	80	1,03

		ERCC6L	complementing rodent repair deficiency, complementation group 6-like									
6550070	NM_005296	GPR23, LPAR4	lysophosphatidic acid receptor 4	323	325	0,99	131	159	0,82	132	125	1,06
2340386	NM_018360	CXorf15	chromosome X open reading frame 15	247	320	0,77	107	130	0,83	110	105	1,04
2070632	NM_020634	GDF3	growth differentiation factor 3	506	298	1,70	86	87	0,98	85	79	1,09
3890372	NM_024094	DCC1, DSCC1	defective in sister chromatid cohesion 1 homolog (S. cerevisiae)	225	296	0,76	105	104	1,01	91	92	0,98
1050088	NM_018228	C14orf115	chromosome 14 open reading frame 115	353	285	1,24	89	90	0,99	83	91	0,91
2710070	NM_024794	ABHD9	abhydrolase domain containing 9	525	96	5,46	97	86	1,13	99	102	0,97
5890170	NM_024865	NANOG	Nanog homeobox	463	77	6,05	88	74	1,19	80	84	0,95
				**1264**	**1506**	**1,35**	**256**	**248**	**1,05**	**118**	**123**	**1,07**
				**1701**	**2263**	**1,29**	**264**	**249**	**0,25**	**61**	**103**	**0,24**

[1] Genes in this table have been classified as specifically expressed in human ES cells in meta-analysis of 38 original human ES cell-transcriptomes (Assou et al., 2007; see Table 4 in this reference). Four genes from this original list of 40 human ES cell-specific genes (*BRRN1*, *KIAA0523*, *MGC3101 and PRO1853*) were absent in our arrays and are not included in our table. Nine genes (*GJA7/GJC1*, *RBM14*, *SLD5*, *RNU3IP2/RRP9*, *TDGF1*, *DNMT3A*, *PWP2*, *DTYMK and MYBL2*) were found not to be differentially expressed among samples included in our study and are also not included. Data are presented as mean gene expression of triplicate samples in relative intensity units. Individual data for each replicate are accessible through Gene Expression Omnibus (GEO) Series accession number GSE17579 (http://www.ncbi.nlm.nih.gov/geo/query/acc.cgi?acc=GSE17579). Data in this table are also presented as ratios of means (iPS/ES, iPS-BC/ES-BC and FH/AH) for individual genes. The symbols of genes that significantly differ (p<0,05; >2-fold difference) in their expression levels between iPS and ES cells are marked with orange colour.

Reference:

1. Assou S, Le Carrour T, Tondeur S, et al. A meta-analysis of human embryonic stem cells transcriptome integrated into a web-based expression atlas. Stem Cells 2007; 25(4):961-973.

Table S13 - Expression of 49 human ES cell-overexpressed genes in undifferentiated human ES and iPS cells, microdissected spontaneously beating clusters derived from human ES (ES-BC) and iPS cells (iPS-BC) as well as human fetal (FH) and adult heart (AH)[1]

Probeset	Access. #	Symbol	Genename	Mean iPS	Mean ES	Ratio iPS/ ES	Mean iPS-BC	Mean ES-BC	Ratio iPS-BC/ ES-BC	Mean FH	Mean AH	Ratio FH/ AH	REF
50204	NM_018189	DPPA4	developmental pluripotency associated 4	10128	10714	0,95	163	172	0,95	85	84	1,01	Assou
6330270	NM_001448	GPC4	glypican 4	4566	9316	0,49	766	809	0,95	352	402	0,87	Assou
4760373	NM_005760	CEBPZ	CCAAT/enhancer binding protein (C/EBP), zeta	4322	7023	0,62	1681	1717	0,98	911	271	3,36	Assou
4390746	NM_006892	DNMT3B	DNA (cytosine-5-)-methyltransferase 3 beta	6976	6133	1,14	219	226	0,97	108	93	1,17	Assou
5050270	NM_020436	SALL4	sal-like 4 (Drosophila)	4405	5366	0,82	502	712	0,71	83	80	1,04	Yu
6960692	NM_032805	ZSCAN10 (ZNF206)	zinc finger and SCAN domain containing 10	6709	4688	1,43	92	91	1,02	80	77	1,04	Yu
4570411	NM_001039591	USP9X	ubiquitin specific peptidase 9, X-linked	2247	4668	0,48	868	1791	0,48	789	403	1,96	Assou
5700070	NM_000478	ALPL	alkaline phosphatase, liver/bone/kidney	4703	4506	1,04	211	307	0,69	625	1177	0,53	Assou

7050682	NM_020796	SEMA6A	sema domain, transmembrane domain (TM), and cytoplasmic domain, (semaphorin) 6A	4510	4448	1,01	1734	1637	1,06	950	426	2,23	Assou
5130156	NM_003106	SOX2	SRY (sex determining region Y)-box 2	3362	4298	0,78	202	381	0,53	91	84	1,09	Assou
5860528	NM_020997	LEFTY1	left-right determination factor 1	934	4172	0,22	97	87	1,12	81	78	1,03	Assou
5700612	NM_012247	SEPHS1	selenophosphate synthetase 1	5409	4016	1,35	1192	989	1,21	936	629	1,49	Assou
60452	NM_015973	GAL	galanin prepropeptide	4644	3945	1,18	224	233	0,96	104	86	1,20	Assou
4880554	NM_005407	SALL2	sal-like 2 (Drosophila)	4134	3808	1,09	758	1213	0,63	413	272	1,52	Yu
4850612	NM_003362	UNG	uracil-DNA glycosylase	3867	3742	1,03	1098	884	1,24	903	1258	0,72	Assou
110437	NM_017489	TERF1	telomeric repeat binding factor (NIMA-interacting) 1	5406	3689	1,47	975	780	1,25	365	232	1,57	Assou
2480291	NM_022806	SNRPN	small nuclear ribonucleoprotein polypeptide N	3010	3374	0,89	718	839	0,86	650	1367	0,48	Assou
5080131	NM_145792	MGST1	microsomal glutathione S-transferase 1	3642	3019	1,21	946	836	1,13	229	1221	0,19	Assou
2070224	NM_004336	BUB1	BUB1 budding uninhibited by benzimidazoles 1 homolog (yeast)	1932	2606	0,74	482	453	1,06	299	84	3,57	Assou
6200168	NM_006875	PIM2	pim-2 oncogene	2075	2526	0,82	192	257	0,75	185	197	0,94	Assou
5310747	NM_033222	PSIP1	PC4 and SFRS1 interacting protein 1	2576	2406	1,07	796	541	1,47	598	182	3,28	Assou

360296	NM_001014809	CRMP1	collapsin response mediator protein 1	2962	2228	1,33	1264	639	1,98	436	169	2,58	Assou
430204	NM_005378	MYCN	v-myc myelocytomatosis viral related oncogene, neuroblastoma derived (avian)	2578	2220	1,16	1677	1246	1,35	427	130	3,30	Lowry
6510762	NM_020873	LRRN1	leucine rich repeat neuronal 1	1709	2200	0,78	167	314	0,53	128	98	1,30	Lowry
150706	NM_006759	UGP2	UDP-glucose pyrophosphorylase 2	1853	2124	0,87	1011	856	1,18	851	620	1,37	Assou
5390669	NM_000814	GABRB3	gamma-aminobutyric acid (GABA) A receptor, beta 3	1896	1783	1,06	129	187	0,69	142	113	1,26	Assou
3120088	NM_001039111	TRIM71, LIN41	tripartite motif-containing 71	1098	1584	0,69	163	279	0,58	85	79	1,08	Yu
5090347	NM_013230	CD24	CD24 molecule	1933	1503	1,29	249	184	1,35	94	86	1,09	Assou
1990661	NM_001010940	C9orf135	chromosome 9 open reading frame 135	3650	1428	2,56	121	100	1,21	94	84	1,12	Yu
4070132	NM_002703	PPAT	phosphoribosyl pyrophosphate amidotransferase	1375	1377	1,00	459	419	1,10	181	117	1,55	Assou
4260682	NM_002482	NASP	nuclear autoantigenic sperm protein (histone-binding)	1690	1338	1,26	594	636	0,93	469	162	2,90	Assou
4560626	NM_007015	LECT1	leukocyte cell derived chemotaxin 1	1115	1268	0,88	114	133	0,86	115	154	0,75	Assou
3130541	NM_001761	CCNF	cyclin F	1126	1219	0,92	419	410	1,02	194	132	1,47	Yu

6330576	NM_001018111	PODXL	podocalyxin-like	916	1147	0,80	518	301	1,72	242	200	1,21	Lowry
70035	NM_174900	ZFP42	zinc finger protein 42 homolog (mouse)	75	1098	0,07	99	94	1,05	85	86	0,99	Yu
7330253	NM_006739	MCM5	minichromosome maintenance complex component 5	1382	1020	1,35	349	246	1,42	247	248	0,99	Assou
4880333	NM_173624	FLJ40504	hypothetical protein FLJ40504	1056	766	1,38	3315	2253	1,47	277	163	1,70	Yu
2970397	NM_145288	ZNF296, ZNF342	zinc finger protein 296	762	762	1,00	113	155	0,73	123	114	1,08	Yu
60280	NM_005763	AASS	aminoadipate-semialdehyde synthase	407	737	0,55	168	220	0,77	193	128	1,51	Assou
130754	NM_002851	PTPRZ1	protein tyrosine phosphatase, receptor-type, Z polypeptide 1	874	718	1,22	113	128	0,88	103	90	1,14	Lowry
630537	NM_018055	NODAL	nodal homolog (mouse)	542	659	0,82	117	130	0,90	89	92	0,96	Lowry
2810192	NM_170678	ITGB1BP3	integrin beta 1 binding protein 3	652	601	1,09	107	198	0,54	3436	16680	0,21	Assou
380259	NM_002164	INDO	indoleamine-pyrrole 2,3 dioxygenase	589	576	1,02	77	91	0,85	95	84	1,13	Assou
3390328	NM_152742	GPC2	glypican 2	497	574	0,87	216	295	0,73	113	94	1,20	Yu
2600465	NM_003026	SH3GL2	SH3-domain GRB2-like 2	563	561	1,00	225	161	1,39	234	441	0,53	Lowry
1660270	NM_005956	MTHFD1	methylenetetrahydrofolate dehydrogenase (NADP+ dependent) 1, methenyltetrahydrofolate cyclohydrolase,	454	382	1,19	154	129	1,19	138	202	0,68	Assou

			formyltetrahydrofolate synthetase										
770632	NM_005454	CER1	cerberus 1, cysteine knot superfamily, homolog (Xenopus laevis)	490	338	1,45	80	79	1,01	78	70	1,12	Yu
6290605	NM_000251	MSH2	mutS homolog 2, colon cancer, nonpolyposis type 1 (E. coli)	336	286	1,17	164	121	1,36	110	90	1,22	Assou
6100215	NM_013264	DDX25	DEAD (Asp-Glu-Ala-Asp) box polypeptide 25	312	235	1,33	122	99	1,23	71	76	0,93	Yu
				**2499**	**2637**	**1,02**	**535**	**511**	**1,02**	**361**	**602**	**1,36**	
				**2102**	**2275**	**0,37**	**607**	**508**	**0,32**	**518**	**2343**	**0,78**	

[1] This table contains selected genes that are classified as overexpressed in human ES cells according to the references listed below. Data are given as mean gene expression of triplicate samples in relative intensity units. Data are also presented as ratios of means (iPS/ES, iiPS-BC/ES-BC and FH/AH) for individual transcripts. Complete data for genes in this table have been deposited in NCBI's Gene Expression Omnibus and are accessible through GEO Series accession number GSE17579 (http://www.ncbi.nlm.nih.gov/geo/query/acc.cgi?acc=GSE17579). The symbols of 2 genes that significantly differ ($p<0,05$; >2-fold difference) in their expression levels between undifferentiated iPS and ES cells are marked with orange colour.

References:

1. Assou S, Le Carrour T, Tondeur S, et al. A meta-analysis of human embryonic stem cells transcriptome integrated into a web-based expression atlas. Stem Cells 2007; 25(4):961-973.

2. Lowry WE, Richter L, Yachechko R, et al. Generation of human induced pluripotent stem cells from dermal fibroblasts. Proc Natl Acad Sci U S A 2008; 105(8):2883-2888.

3. Yu J, Vodyanik MA, Smuga-Otto K, et al. Induced pluripotent stem cell lines derived from human somatic cells. Science 2007; 318(5858):1917-1920.

Table S14 - Expression of 83 cardiac enriched genes in undifferentiated human ES and iPS cells, microdissected beating clusters derived from human ES cells (ES-BC) and iPS cells (iPS-BC) as well as human fetal (FH) and adult heart (AH)[1]

Probe set	Symbol	Gene name	Accession #	Mean hiPS	Mean hES	Ratio hiPS/ hES	Mean hiPS-BC	Mean hES-BC	Ratio hiPS-BC/ hES-BC	Mean FH	Mean AH	Ratio FH/ AH	REF
4290333	MYL2	myosin, light chain 2, regulatory, cardiac, slow	NM_000432	127	89	1,43	643	969	0,66	44246	48053	0,92	S
4920561	MB	myoglobin	NM_005368	104	113	0,92	852	1995	0,43	31903	48023	0,66	S
6270739	NPPA	natriuretic peptide precursor A	NM_006172	90	91	0,99	20174	16185	1,25	50322	46648	1,08	C, Mi
1440181	MYH7	myosin, heavy chain 7, cardiac muscle, beta	NM_000257	97	98	0,99	16528	16869	0,98	41487	40903	1,01	S,B
1500349	MYH6	myosin, heavy chain 6, cardiac muscle, alpha	NM_002471	135	113	1,19	36161	45001	0,8	35399	38194	0,93	dB
3890603	MYL3	myosin, light chain 3, alkali; ventricular, skeletal, slow	NM_000258	129	83	1,55	5334	12316	0,43	24957	37116	0,67	B
4260201	ACTC1	actin, alpha, cardiac muscle 1	NM_005159	1070	1276	0,84	22395	25960	0,86	26583	29645	0,90	S
6940768	MYBPC3	myosin binding protein C, cardiac	NM_000256	86	86	1	6527	12234	0,53	14499	26596	0,55	S
1580592	CKM	creatine kinase, muscle	NM_001824	86	81	1,06	848	2192	0,39	16315	23530	0,69	Sri

2690343	TNNC1	troponin C type 1 (slow)	NM_003280	222	124	1,79	4689	7601	0,62	17232	22693	0,76	S
160692	MYOM1	myomesin 1, 185kDa	NM_003803	92	79	1,16	8571	14388	0,6	21824	20040	1,09	S
7650296	DCN	decorin	NM_133505	79	84	0,94	15098	4222	3,58	16997	19300	0,88	S
1580037	HRC	histidine rich calcium binding protein	NM_002152	146	116	1,26	1358	2245	0,6	8374	18035	0,46	S,B, A
4010039	TNNT2	troponin T type 2 (cardiac)	NM_000364	110	113	0,97	7545	13228	0,57	13152	17868	0,74	V
770079	ATP2A2	ATPase, Ca++ transporting, cardiac muscle, slow twitch 2 (SERCA2)	NM_170665	825	759	1,09	6695	6437	1,04	13203	17380	0,76	S
4560523	HSPB7	heat shock 27kDa protein family, member 7 (cardiovascular)	NM_014424	88	83	1,06	1924	3590	0,54	8270	16392	0,50	S,B
1510612	MYL4	myosin, light chain 4, alkali; atrial, embryonic	NM_002476	285	131	2,18	19346	26011	0,74	29122	14220	2,05	C
1850082	SYNPO2L	synaptopodin 2-like	NM_024875	80	97	0,82	6156	7425	0,83	7393	12831	0,58	S
1240300	ENO3	enolase 3 (beta, muscle)	NM_001976	409	541	0,76	2471	8083	0,31	9635	11430	0,84	B
2070630	MYL7	myosin, light chain 7, regulatory	NM_021223	278	281	0,99	20813	22847	0,91	19197	10450	1,84	S
3400538	IGFBP7	insulin-like growth factor binding protein 7	NM_001553	119	110	1,08	2264	627	3,61	5338	8329	0,64	S
3390735	ADPRHL1	ADP-ribosylhydrolase like 1	NM_199162	102	106	0,96	1034	987	1,05	4614	8258	0,56	S
6560577	HSPB3	heat shock 27kDa protein 3	NM_006308	94	88	1,07	997	1775	0,56	7291	7271	1,00	S, Mi

1430487	MGP	matrix Gla protein	NM_000900	80	79	1,01	1570	377	4,16	3654	6480	0,56	Mi
3140520	TMOD1	tropomodulin 1	NM_003275	110	133	0,83	2032	3084	0,66	5743	6077	0,95	Mi
6250192	COL6A3	collagen, type VI, alpha 3	NM_057165	129	133	0,97	4151	998	4,16	8047	4652	1,73	Mo
5360494	SMYD1	SET and MYND domain containing 1	NM_198274	65	74	0,88	2198	3710	0,59	5819	4196	1,39	S, T
670196	ACTN2	actinin, alpha 2	NM_001103	138	123	1,12	1278	1532	0,83	4173	4114	1,01	S
1660241	RYR2	ryanodine receptor 2 (cardiac)	NM_001035	119	130	0,92	909	1106	0,82	3141	3958	0,79	S
3890743	APOBEC2	apolipoprotein B mRNA editing enzyme, catalytic polypeptide-like 2	NM_006789	83	93	0,89	979	2299	0,43	3341	3663	0,91	Mc
4070025	POPDC2	popeye domain containing 2	NM_022135	82	79	1,04	1232	1506	0,82	2976	3014	0,99	S,B, Bre
6020132	CLIC5	chloride intracellular channel 5	NM_016929	95	100	0,95	337	593	0,57	643	2801	0,23	vTin
3420035	HAND1	heart and neural crest derivatives expressed 1	NM_004821	106	98	1,08	13537	3961	3,42	3044	2751	1,11	C
4120554	SMPX	small muscle protein, X-linked	NM_014332	77	81	0,95	1124	1243	0,9	1906	2108	0,90	S
770669	CSRP3	cysteine and glycine-rich protein 3 (cardiac LIM protein)	NM_003476	77	73	1,05	882	1176	0,75	2358	1775	1,33	S
2370438	A2M	alpha-2-macroglobulin	NM_000014	76	88	0,86	496	689	0,72	1746	1609	1,09	Mi
7040497	ART3	ADP-ribosyltransferase 3	NM_001179	98	101	0,97	271	319	0,85	1330	1591	0,84	A

7210349	BVES	blood vessel epicardial substance	NM_147147	168	210	0,8	2621	3095	0,85	5144	1549	3,32	Mi
2260646	PKP2	Plakophilin 2	NM_001005242	300	252	1,19	1892	2378	0,8	2004	1534	1,31	vTin
2490364	LUM	lumican	NM_002345	90	84	1,07	12152	1050	11,57	3134	1485	2,11	Mi
7150348	NKX2-5	NK2 transcription factor related, locus 5	NM_004387	82	115	0,71	635	694	0,91	1261	1420	0,89	B
3850246	HOPX	HOP homeobox	NM_139212	74	80	0,93	418	248	1,69	2909	1414	2,06	S, Che
2120021	COL3A1	collagen, type III, alpha 1	NM_000090	89	87	1,02	24651	8823	2,79	15797	1378	11,46	S,B
3190278	LDB3	LIM domain binding 3	NM_007078	91	86	1,06	480	718	0,67	885	1362	0,65	S,B
3310022	IRX5	iroquois homeobox 5	NM_005853	161	151	1,07	581	1108	0,52	1173	1344	0,87	B
2750187	NEXN	nexilin (F actin binding protein)	NM_144573	169	171	0,99	1445	1296	1,11	1625	1283	1,27	B
6250128	JPH2	Junctophilin 2	NM_020433	83	83	1	277	380	0,73	901	1243	0,72	B
160433	SRD5A2L2	steroid 5 alpha-reductase 2-like 2	NM_001010874	77	86	0,9	771	3632	0,21	1059	1153	0,92	S
5090750	FOXC1	forkhead box C1	NM_001453	201	119	1,69	1853	2298	0,81	1327	1133	1,17	Mi
5910632	SMARCD3	SWI/SNF related, matrix associated, actin dependent regulator of chromatin, subfamily d, member 3 (Baf60c)	NM_003078	144	123	1,17	1305	1036	1,26	988	1082	0,91	S,B, Lic
5670441	FSD2	fibronectin type III and SPRY domain containing 2	NM_001007122	80	85	0,94	431	732	0,59	1682	1062	1,58	Mi

4860681	GUCY1A3	guanylate cyclase 1, soluble, alpha 3	NM_000856	102	86	1,19	3517	1675	2,1	1858	1042	1,78	Ord
4250689	PLN	phospholamban	NM_002667	101	109	0,93	1125	1913	0,59	1965	1012	1,94	S
3940484	MEF2D	myocyte enhancer factor 2D	NM_005920	210	200	1,05	464	632	0,73	705	866	0,81	S
2490026	MYLK3	myosin light chain kinase 3, cardiac MLCK	NM_182493	82	81	1,01	267	514	0,52	528	845	0,62	S
6960487	KBTBD10	kelch repeat and BTB (POZ) domain containing 10	NM_006063	82	88	0,93	531	650	0,82	880	816	1,08	Mi
6590538	TF	transferrin	NM_001063	101	85	1,19	5296	696	7,61	164	776	0,21	Mi
4010132	MYH7B	myosin, heavy chain 7B, cardiac muscle, beta	NM_020884	92	87	1,06	189	297	0,64	666	719	0,93	Kou
160445	RGS5	regulator of G-protein signaling 5	NM_003617	134	136	0,99	1323	1279	1,03	1739	714	2,44	C
620056	MYOZ2	myozenin 2 (calsarcin 1)	NM_016599	82	83	0,99	258	164	1,57	748	624	1,20	C, Cos
2360753	TBX2	T-box 2	NM_005994	84	90	0,93	484	1321	0,37	491	466	1,05	Mi, C
380561	NEBL	nebulette	NM_006393	105	171	0,61	1278	1661	0,77	1056	435	2,43	S
4490673	TWIST1	twist homolog 1 (Drosophila)	NM_000474	227	216	1,05	591	640	0,92	376	426	0,88	Lim
3440239	GATA4	GATA binding protein 4	NM_002052	110	101	1,09	395	568	0,7	365	410	0,89	S
4640563	HAND2	heart and neural crest derivatives expressed 2	NM_021973	99	87	1,14	348	314	1,11	452	338	1,34	C

2490564	SLC8A1	solute carrier family 8 (sodium/calcium exchanger, NCX1), member 1	AK095013	86	77	1,12	605	1003	0,6	1161	285	4,07	S
1710142	RGS4	regulator of G-protein signaling 4	NM_005613	85	83	1,02	853	1219	0,7	420	240	1,75	Pra
4040162	MEF2C	myocyte enhancer factor 2C	NM_002397	88	83	1,06	394	858	0,46	551	234	2,35	Kim
6250403	GATA5	GATA binding protein 5	NM_080473	101	85	1,19	554	911	0,61	195	172	1,13	Uch
3440685	TTN	titin	NM_133378	94	89	1,06	548	1152	0,48	279	171	1,63	Che
3520246	CXCL14	chemokine (C-X-C motif) ligand 14	NM_004887	88	96	0,92	376	195	1,93	105	141	0,74	C
6380484	SERPINA1	serpin peptidase inhibitor, clade A (alpha-1 antiproteinase, antitrypsin), member 1	NM_001002235	78	84	0,93	931	1416	0,66	141	136	1,04	Mi, C
2030709	TTR	transthyretin	NM_000371	241	95	2,54	3449	4017	0,86	122	127	0,96	S
2140653	TPM1	tropomyosin 1 (alpha)	NM_001018008	175	202	0,87	466	792	0,59	182	116	1,57	S
3420682	IGF2	insulin-like growth factor 2 (somatomedin A)	NM_001007139	87	81	1,07	225	324	0,69	159	107	1,49	C
7210240	FGB	fibrinogen beta chain	NM_005141	130	96	1,35	3807	2416	1,58	100	106	0,94	S
2320377	AHSG	alpha-2-HS-glycoprotein	NM_001622	83	94	0,88	784	1142	0,69	104	95	1,09	Mi
4760553	ISL1	ISL LIM homeobox 1	NM_002202	291	123	2,37	2309	2351	0,98	106	92	1,15	S
4810209	GABRP	gamma-aminobutyric acid (GABA)	NM_014211	105	120	0,88	1662	236	7,04	96	90	1,07	Fri

		A receptor, pi										
2030161	AFP	alpha-fetoprotein	NM_001134	261	211	1,24	12348	11968	1,03	96	87	1,10	Seg
5910398	NPNT	nephronectin	NM_001033047	83	80	1,04	361	197	1,83	171	81	2,11	S,B
5810634	TDO2	tryptophan 2,3-dioxygenase	NM_005651	72	72	1	273	108	2,53	73	77	0,95	S
2230008	FGG	fibrinogen gamma chain	NM_000509	92	78	1,18	2059	1822	1,13	105	76	1,38	C
			Mean	**143**	**136**	**1,08**	**4060**	**4189**	**1,32**	**6883**	**7498**	**1,29**	
			SD	**143**	**157**	**0,31**	**6694**	**7208**	**1,70**	**10930**	**12178**	**1,29**	

[1] Genes in this table have been reported to be specific or enriched in the heart, isolated cardiomyocytes or cardiac precursor cells and/or microdissected cardiac clusters derived from human ES cells (see references below). Data are presented as mean gene expression of triplicate samples in relative intensity units. Expression levels of all transcripts were comparable between iPS-BCs and ES-BCs (p>0,05); in cases, such as HAND1, TBX2 or MYL3, the mean values strongly differ, but the difference does not reach statistical significance because of variable expression of these transcript in triplicate samples. Data are also presented as ratios of means (hiPS/hES, hiPS-BC/hES-BC, FH/AH) for individual transcripts. Complete data for genes in this table have been deposited in NCBI's Gene Expression Omnibus and are accessible through GEO Series accession number GSE17579 (http://www.ncbi.nlm.nih.gov/geo/query/acc.cgi?acc=GSE17579). The symbols of 8 genes that significantly differ (p<0,05; >2-fold difference) in their expression levels between iPS-BCs and ES-BCs are marked with orange boxes.

References:

A - Arvanitis, DA et al. Histidine-rich Ca-binding protein interacts with sarcoplasmic reticulum Ca-ATPase**.** Am J Physiol Heart Circ Physiol 293(3): H1581-H1589, 2007.

B - Beqqali A et al. Genome-wide transcriptional profiling of human embryonic stem cells differentiating to cardiomyocytes. Stem Cells 24: 1956-1967, 2006.

Bre - Breher SS, et al. Popeye domain containing gene 2 (Popdc2) is a myocyte-specific differentiation marker during chick heart development. Dev Dyn. 229(3):695-702, 2004.

dB - de Bold A. Atrial natriuretic factor: a hormone produced by the heart. Science 230 (4727): 767–70, 1985.

C – Cao F et al. Transcriptional and functional profiling of human embryonic stem cell-derived cardiomyocytes. PLOSone 3 (10): e3474, 2008.

Che - Chen F, et al. Hop is an unusual homeobox gene that modulates cardiac development. Cell 110(6):713-723, 2002.

Cos - Costantini DL, et al. The homeodomain transcription factor Irx5 establishes the mouse cardiac ventricular repolarization gradient. Cell 123(2):347-358, 2005.

Fri - Friedrich M, et al. Genomic organization and expression of the human mono-ADP-ribosyltransferase ART3 gene. Biochim Biophys Acta 1759(6):270-280, 2006.

Kim - Kim Y, et al. The MEF2D transcription factor mediates stress-dependent cardiac remodeling in mice. J Clin Invest. 118(1):124-132, 2008

Kou - Koushik SV, et al. Targeted inactivation of the sodium-calcium exchanger (Ncx1) results in the lack of a heartbeat and abnormal myofibrillar organization. Faseb J 15(7):1209-1211, 2001.

Lic - Lickert H, et al. Baf60c is essential for function of BAF chromatin remodelling complexes in heart development. Nature 432(7013):107-112, 2004.

Lim - Lim D.S., et al. Expression profiling of cardiac genes in human hypertrophic cardiomyopathy: insight into the pathogenesis of phenotypes. J. Am. Coll. Cardiol. 38 (4), 1175-1180, 2001.

Mc - McKeown CR, et al. Tropomodulin1 is required in the heart but not the yolk sac for mouse embryonic development. Circ Res. 103(11):1241-1248, 2008.

Mi – Miller RA et al. Efficient array-based identification of novel cardiac genes through differentiation of mouse ESCs. PLOSone 3 (5): e2176, 2008.

Mo - Moretti A, et al. Multipotent embryonic isl1+ progenitor cells lead to cardiac, smooth muscle, and endothelial cell diversification. Cell 127:1151-65, 2006.

Ord - Ordway GA, and Garry DJ. Myoglobin: an essential hemoprotein in striated muscle. J Exp Biol. 207(Pt 20):3441-3446, 2004.

Pra - Prabhakar R, et al. A mouse model of familial hypertrophic cardiomyopathy caused by a alpha-tropomyosin mutation. Mol Cell Biochem 251: 33–42, 2003.

S – Synnergren J et al. Molecular signature of cardiomyocyte clusters derived from human embryonic stem cells. Stem Cells 26: 1831-1840, 2008.

Seg - Seguchi O, et al. A cardiac myosin light chain kinase regulates sarcomere assembly in the vertebrate heart. J Clin Invest 117(10):2812-2824, 2007.

Sri - Srivastava D. HAND proteins: molecular mediators of cardiac development and congenital heart disease. Trends Cardiovasc. Med. 9 (1-2): 11–8, 1999.

T – Tan X et al. Smyd1, a histone methyltransferase, is required for myofibril organization and muscle contraction in zebrafish embryos. PNAS USA 103:2713-2718, 2006.

Uch - Uchida S, et al. An integrated approach for the systematic identification and characterization of heart-enriched genes with unknown functions. BMC Genomics. 10:100, 2009.

V - Vangheluwe P, et al. Modulating sarco(endo)plasmic reticulum Ca2+ ATPase 2 (SERCA2) activity: cell biological implications. Cell Calcium. 38(3-4):291-302, 2005.

vTin - van Tintelen JP, et al. Plakophilin-2 mutations are the major determinant of familial arrhythmogenic right ventricular dysplasia/cardiomyopathy. Circulation 113(13): 1650–1658, 2006.
