# Supplementary material for: Global transcriptional profiles of beating clusters derived from human induced pluripotent stem cells and embryonic stem cells are highly similar
Source: BMC Dev Biol. 2010 Sep 15;10:98. doi: 10.1186/1471-213X-10-98 (PMC2946283; doi:10.1186/1471-213X-10-98)
Supplement: Additional file 7 — qRT-PCR validation of microarray data with Human ES cell StellArray qPCR Array (Lonza). This file is a PDF document containing Table S15 showing the results of qRT-PCR analysis of expression of selected pluripotency and differentiations genes in undifferentiated human ES and iPS cells in comparison with microarray data obtained in this study. [file 1471-213X-10-98-S7.PDF]

Table S15 - qRT-PCR analysis of expression of 95 pluripotency and differentiations genes in undifferentiated ES cells and iPS cells using Human embryonic stem cell StellARray™ qPCR array (Lonza) and comparison with microarray data.

			StellArray		Microarray		StellArray, ct values			StellArray, ct values		
Rank	Gene Name	Gene category	p-Value	Fold Change (iPS/ES)	p-Value*	Fold Change (iPS/ES)	HES2, p97	HES2, p132	HES2, p129	hiPS, p28	hiPS, p23	hiPS, p20
**1**	**ZFP42**	pluripotency	**0.0006**	**-3170.21**	**0.0118**	**-13.97**	**24.0**	**27.2**	**26.4**	**35.8**	**37.3**	**39.2**
**2**	**H19**	visceral endoderm	**0.0034**	**21.60**	**0.0328**	1.55	**32.9**	**33.8**	**34.6**	**28.2**	**29.6**	**28.6**
**3**	**TTR**	visceral endoderm	**0.0117**	**114.45**	**0.1378**	**2.53**	**34.2**	**35.1**	**35.7**	**27.4**	**27.3**	**30.5**
**4**	**HPRT1**	pluripotency	**0.0484**	**-2.53**	**0.0214**	**-2.39**	**24.4**	**26.9**	**26.6**	**26.3**	**27.4**	**27.0**
5	COL1A1	pluripotency	0.0749	-2.48	0.1807	-1.36	23.7	25.6	24.4	25.4	25.5	25.7
6	PIM2	pluripotency	0.0767	-1.95	0.3139	-1.03	24.1	25.9	25.2	25.2	26.5	25.3
7	NEUROD1	neuroectoderm	0.0853	-2.76	0.1870	-1.06	35.2	37.0	37.4	36.4	38.3	38.4
8	FN1	pluripotency	0.0976	2.53	0.7358	-1.05	24.5	25.5	24.7	23.3	23.5	22.6
9	GDF3	pluripotency	0.1329	2.12	0.0539	1.70	27.7	29.1	28.0	26.3	27.9	26.2
10	MED17	pluripotency	0.1686	1.43	0.5558	1.09	27.2	28.7	28.1	26.6	27.8	26.8
11	NAT1	pluripotency	0.1687	2.12	0.0647	-1.06	29.7	30.3	30.5	29.0	29.1	27.9
12	SOX2	pluripotency	0.1696	-1.40	0.3280	-1.59	25.4	27.1	26.5	26.2	26.9	26.3
13	WNT5A	differentiation	0.1828	2.74	0.5990	1.08	31.8	33.4	35.2	30.1	33.0	31.3
14	FOXH1	pluripotency	0.1835	1.77	0.2891	1.09	33.0	35.4	35.0	33.5	33.6	32.8
15	SOX17	definitive endoderm	0.1961	2.93	0.2447	1.64	34.9	31.7	31.6	30.0	29.9	29.4
16	PARD6A	keratinocyte	0.1965	1.32	0.5896	-1.00	28.9	31.5	31.1	29.2	30.4	29.4
17	DNMT3B	pluripotency	0.1978	1.28	0.4518	1.14	20.5	22.2	21.9	20.3	21.4	20.7
18	TRIM71	pluripotency	0.1985	-1.31	0.6258	1.06	23.1	24.7	24.3	23.5	24.5	24.0
19	UTF1	pluripotency	0.2061	1.37	0.2536	1.27	29.9	31.5	31.4	29.6	30.4	30.2
20	GABRB3	pluripotency	0.2139	1.75	0.9449	0.99	27.2	29.8	28.3	26.4	28.5	26.8
21	GSC	visceral and definitive endoderm	0.2214	-2.46	0.3050	-1.21	28.0	32.0	29.6	30.2	32.1	29.7
22	TERT	pluripotency	0.2222	-1.61	0.8305	-1.05	28.1	28.8	29.0	28.3	29.4	28.9
23	IL6ST	pluripotency	0.2317	1.72	0.9838	-1.13	27.1	30.7	29.3	26.9	29.0	27.3
24	ZSCAN10	pluripotency	0.2330	1.23	0.0984	1.43	24.6	26.2	25.3	24.3	25.1	24.6
25	PTEN	pluripotency	0.2331	1.26	0.6235	1.06	26.5	29.0	28.3	27.0	27.6	27.0
26	EOMES	pluripotency	0.2393	2.23	0.2227	1.76	30.6	30.2	28.9	28.0	27.8	28.3
27	CCND1	Keratinocyte	0.2433	1.62	0.0398	1.80	23.7	25.7	24.8	24.0	23.8	23.4
28	T	Mesoderm	0.2450	2.87	0.3696	-1.17	38.1	35.2	35.2	34.1	33.0	33.6
29	DPPA3	pluripotency	0.2454	-1.47	0.6032	-1.03	29.4	31.4	31.1	29.9	31.1	32.0
30	AKAP13	pluripotency	0.2467	-1.18	0.3109	-1.02	26.9	29.3	28.6	27.8	28.7	27.8
31	PIPOX	pluripotency	0.2488	2.93	0.0336	1.40	26.7	29.2	29.3	24.8	28.2	27.5
32	POU5F1	pluripotency	0.2514	-1.26	0.1996	-1.77	20.9	23.2	23.1	21.6	22.8	22.3
33	LEFTY2	pluripotency	0.2532	-1.87	0.3038	-1.90	26.9	28.9	26.3	29.6	31.1	25.9

34	BGLAP	Keratinocyte	0.2547	1.35	0.5989	-1.02	28.8	30.4	30.4	29.0	29.2	29.1
35	VASH2	pluripotency	0.2627	-1.11	0.6680	-1.59	24.8	26.4	26.1	25.1	26.4	25.2
36	REST	pluripotency	0.2658	-1.03	0.0366	-1.39	25.4	27.2	26.9	25.8	26.8	25.8
37	NES	Neurectoderm	0.2665	-1.25	0.8162	-1.02	26.6	28.2	28.3	27.3	28.3	27.1
38	FGF2	pluripotency	0.2669	-1.36	0.5896	-1.24	23.4	26.1	25.7	24.3	25.9	25.0
39	GRB7	pluripotency	0.2689	-1.22	0.8740	1.02	26.3	28.2	27.9	27.1	28.3	26.8
40	DPPA4	pluripotency	0.2700	1.06	0.5779	-1.11	21.4	23.9	23.0	21.8	22.9	22.0
41	FLT1	pluripotency	0.2719	-1.14	0.7559	1.02	27.1	29.5	29.2	27.8	29.0	28.2
42	IFITM1	pluripotency	0.2760	-1.72	0.1470	-1.86	23.3	23.8	22.8	23.7	24.2	22.8
43	LOX	Fibroblast	0.2799	-1.60	0.8428	-1.13	28.1	29.2	27.8	27.9	29.3	28.3
44	CD9	pluripotency	0.2812	-1.32	0.8877	-1.11	23.7	25.5	25.1	23.9	26.3	24.5
45	LIN28	pluripotency	0.2819	1.06	0.9746	-1.10	20.4	22.9	22.8	21.1	22.1	21.2
46	Hs18s	Housekeeping	0.2834	1.01	ND	ND	10.4	11.7	11.1	10.3	11.0	10.5
47	TP53	pluripotency	0.2849	1.02	0.3168	1.42	23.3	25.0	24.1	23.5	24.4	23.3
48	SLC16A1	pluripotency	0.2931	-1.04	0.3913	0.93	21.4	24.0	23.2	22.1	23.2	22.2
49	ACTC1	pluripotency	0.2970	-1.13	0.6936	1.04	17.0	18.9	18.8	18.1	18.1	17.9
50	TUBB3	Keratinocyte	0.3008	-1.33	0.7216	-1.08	23.5	25.8	26.0	24.4	26.1	24.7
51	GAL	pluripotency	0.3089	-1.11	0.5066	1.18	23.6	25.9	24.7	24.3	25.2	23.9
52	FOXD3	pluripotency	0.3139	1.82	0.2709	1.24	33.5	35.0	37.6	32.5	35.1	33.7
53	MYST2	Keratinocyte	0.3145	1.13	0.2168	-1.10	24.4	26.2	25.2	23.8	25.5	24.8
54	DPPA2	pluripotency	0.3147	1.30	0.7426	-1.16	27.9	29.8	29.4	28.2	28.2	28.7
55	DCN	Fibroblast	0.3170	1.26	0.1681	-1.02	33.3	33.1	33.6	32.3	32.7	32.2
56	TDGF1	pluripotency	0.3208	1.24	0.5951	1.04	24.2	26.1	26.3	24.6	24.8	25.1
57	COL2A1	pluripotency	0.3230	4.25	0.3000	-1.13	30.8	33.3	32.5	30.5	29.0	32.2
58	CCNA2	Keratinocyte	0.3262	-1.08	0.5547	1.19	23.2	25.4	25.4	23.4	25.2	24.6
59	LIFR	Differentiation	0.3262	1.05	0.9579	-1.14	26.9	29.0	28.4	27.4	27.7	28.0
60	IGF2	Differentiation	0.3324	-1.59	0.2012	-1.00	30.8	32.4	30.4	30.5	32.3	31.1
61	GJA1	Keratinocyte	0.3331	-1.07	0.0188	-1.78	21.3	23.6	23.7	21.6	23.3	22.7
62	GATA6	Differentiation	0.3370	1.90	0.1924	-1.17	32.2	30.0	29.0	29.1	28.9	27.2
63	LAMA1	pluripotency	0.3442	1.05	0.6292	-1.16	27.6	28.5	28.3	27.9	28.0	27.0
64	GBX2	pluripotency	0.3451	-1.29	0.1324	-1.10	30.3	31.1	31.6	30.5	30.8	32.1
65	DPPA5	pluripotency	0.3482	1.40	0.9085	1.02	31.9	36.8	34.4	32.7	34.2	32.4
66	PAX6	Differentiation	0.3492	-1.58	0.9325	1.01	30.3	30.2	30.1	32.7	30.0	29.9
67	CXCR4	Differentiation	0.3593	1.47	0.6719	1.19	30.6	30.6	27.2	27.6	29.0	26.2
68	CRABP2	pluripotency	0.3650	1.40	0.5510	-1.01	23.5	24.0	24.0	23.4	22.7	23.0
69	MYC	Reprogramming	0.3694	-1.52	0.1577	0.67	24.7	27.6	25.1	25.2	27.1	25.3
70	NOTCH1	Fibroblast	0.3787	-1.32	0.3820	-1.29	30.1	30.2	31.0	30.4	30.7	29.7
71	SYP	pluripotency	0.3811	-1.43	0.3600	1.32	29.2	32.7	32.4	30.1	33.1	31.5
72	ATL1	Fibroblast	0.3911	1.00	0.6022	-1.07	27.1	30.1	29.8	27.6	29.9	28.3
73	AFP	Differentiation	0.4000	1.05	0.4155	1.24	27.1	30.1	30.3	27.4	29.5	28.9
74	PODXL	pluripotency	0.4010	1.29	0.8342	-1.08	22.7	24.5	24.0	23.7	23.8	22.0
75	SHH	Differentiation	0.4017	1.52	0.4602	-1.01	35.0	32.2	31.6	31.4	32.2	29.9
76	CDKN1C	pluripotency	0.4063	-1.02	0.4778	-1.21	28.7	30.2	30.6	30.1	29.2	29.6
77	MME	Keratinocyte	0.4099	1.03	0.5284	-1.12	26.5	29.3	28.2	26.3	28.5	28.3

78	FGF4	pluripotency	0.4158	-1.12	0.7511	0.94	30.8	32.0	30.8	32.0	32.3	29.6
79	NANOG	pluripotency	0.4196	-1.12	0.0005	6.05	24.0	26.5	24.6	24.5	26.4	23.8
80	KLF4	Reprogramming	0.4282	-1.18	0.9591	1.01	28.2	30.8	31.5	29.4	30.6	29.4
81	GATA4	Differentiation	0.4406	1.18	0.3524	1.08	33.4	30.1	28.4	29.1	28.6	28.4
82	LEFTY1	pluripotency	0.4487	1.07	0.3461	0.22	28.3	32.2	27.2	32.0	34.4	26.0
83	NCAM1	Differentiation	0.4502	-1.28	0.0677	-1.14	29.7	29.1	29.3	28.1	29.9	29.3
84	FOXA2	Differentiation	0.4530	-1.03	0.1497	1.07	33.4	28.0	28.9	27.8	28.4	27.7
85	CYLD	Fibroblast	0.4615	-1.04	0.6739	0.96	27.6	31.4	29.5	28.3	29.2	29.8
86	ABCG2	pluripotency	0.4638	-2.40	0.6624	0.95	31.6	35.2	37.3	34.0	34.5	37.6
87	IGFBP5	Fibroblast	0.4659	-2.99	0.4565	-1.25	30.7	28.5	26.4	30.1	29.5	27.3
88	NODAL	pluripotency	0.5299	-1.51	0.7271	0.82	27.0	30.8	27.0	27.7	29.4	27.1
89	COMMD3	pluripotency	0.5496	2.22	0.3810	1.12	24.8	31.8	30.1	27.5	30.1	24.2
90	CER1	Differentiation	0.5547	-1.18	0.6051	1.45	25.1	25.7	23.0	24.2	26.2	22.5
91	RUNX1	Differentiation	0.5734	1.24	0.0973	-1.26	34.4	40.0	34.4	36.6	34.6	33.8
**92**	**HSGenomic**	**Control**	**NA**	**NA**	**NA**	**NA**	**38.0**	**40.0**	**38.7**	**40.0**	**37.3**	**37.1**
93	CDX2	Differentiation	NA	NA	0.5988	-1.03	36.3	40.0	35.4	35.4	33.8	39.1
94	COL3A1	Fibroblast	NA	NA	0.8667	1.02	38.8	36.1	35.0	36.4	39.4	39.6
95	FBXO15	pluripotency	NA	NA	0.0573	1.43	40.0	40.0	35.1	39.7	35.6	33.7
96	PECAM1	Mesoderm	NA	NA	0.7684	-1.16	35.1	40.0	35.4	32.9	33.2	40.0

*Cells marked in blue indicate the genes for which the results of microarray and qRT-PCR analyses differed (8 out of 90 successfully amplified genes, 8.9%). Orange – housekeeping gene used for normalization. Red – Human genomic DNA control. ND – not determined; NA – not applicable.
