# Supplementary material for: Global transcriptional profiles of beating clusters derived from human induced pluripotent stem cells and embryonic stem cells are highly similar
Source: BMC Dev Biol. 2010 Sep 15;10:98. doi: 10.1186/1471-213X-10-98 (PMC2946283; doi:10.1186/1471-213X-10-98)
Supplement: Additional file 9 — Gene ontology analysis of genes upregulated in iPS-BCs and ES-BCs as compared to their respective undifferentiated counterparts. This file is a PDF document containing Table S20 with the list of all overrepresented GO terms in iPS-BCs compared to iPS cells and in ES-BCs compared to ES cells. [file 1471-213X-10-98-S9.PDF]

Table S20 - Gene ontology analysis of genes upregulated in iPS-BCs and ES-BCs as compared to their respective undifferentiated counterparts. Only significantly enriched GO-terms are shown (p<0.001). nd – not detected as significantly enriched.

Biological process

Term	Count		p-value	
	iPS-BC	ES-BCs	iPS-BCs	ES-BCs
organ morphogenesis	115	73	1.2E-31	2.6E-16
tissue development	116	73	5.8E-26	6.8E-13
vasculature development	63	37	3.6E-22	3.9E-10
blood vessel development	61	36	2.8E-21	7.8E-10
heart development	53	41	3.0E-18	7.8E-15
muscle organ development	52	42	6.5E-18	7.2E-16
heart morphogenesis	29	26	6.7E-16	2.8E-16
tissue morphogenesis	44	29	3.4E-15	5.1E-9
blood vessel morphogenesis	48	27	3.9E-15	2.2E-6
muscle tissue development	35	28	6.7E-14	5.3E-12
enzyme linked receptor protein signaling pathway	60	41	2.5E-13	1.9E-8
striated muscle tissue development	33	26	5.2E-13	6.3E-11
bone development	32	21	7.7E-12	4.6E-7
regulation of cell migration	36	21	1.4E-10	6.3E-5
cartilage development	23	12	2.8E-10	4.1E-4
skeletal system morphogenesis	28	17	5.2E-10	3.5E-5
lung development	26	15	6.3E-10	1.1E-4
respiratory tube development	26	15	1.3E-9	1.5E-4
cardiac muscle tissue development	19	17	3.9E-9	2.1E-9
chordate embryonic development	50	33	5.8E-9	2.8E-5
branching morphogenesis of a tube	20	nd	6.2E-9	-
regulation of blood vessel size	18	10	8.3E-9	4.9E-4
kidney development	24	nd	1.1E-8	-
regulation of heart contraction	21	17	1.8E-8	1.8E-7
cardiac muscle tissue morphogenesis	13	14	1.9E-8	2.7E-11
muscle tissue morphogenesis	13	14	1.9E-8	2.7E-11
positive regulation of transcription, DNA-dependent	62	42	2.8E-8	3.8E-5
positive regulation of RNA metabolic	62	42	3.8E-8	4.6E-5

process				
mesenchyme development	17	nd	4.4E-8	-
positive regulation of cell proliferation	55	nd	9.3E-8	-
cell migration	42	28	1.1E-7	1.1E-4
metanephros development	15	nd	1.9E-7	-
mesenchymal cell development	16	nd	2.3E-7	-
mesenchymal cell differentiation	16	nd	2.3E-7	-
positive reg. of nucleobase, nucleoside, nucleotide and nucleic acid metabolic process	71	52	4.9E-7	1.8E-5
positive regulation of transcription	66	47	5.0E-7	4.9E-5
embryonic organ development	30	19	5.8E-7	6.6E-4
positive reg. of nitrogen compound metabolic process	72	52	7.9E-7	4.1E-5
angiogenesis	27	nd	8.7E-7	-
muscle cell development	16	18	8.7E-7	1.6E-10
positive regulation of gene expression	66	47	1.4E-6	9.7E-5
positive regulation of biosynthetic process	75	55	1.6E-6	3.9E-5
smooth muscle contraction	12	nd	1.7E-6	-
negative regulation of cell proliferation	47	31	1.8E-6	7.6E-4
striated muscle cell development	15	17	1.9E-6	4.5E-10
positive regulation of cellular biosynthetic process	74	55	1.9E-6	2.8E-5
morphogenesis of an epithelium	21	nd	2.2E-6	-
neural crest cell development	12	nd	2.4E-6	-
neural crest cell differentiation	12	nd	2.4E-6	-
striated muscle contraction	14	13	2.4E-6	5.3E-7
positive reg. of macromolecule biosynthetic process	71	51	2.7E-6	1.1E-4
negative regulation of gene expression	58	42	4.2E-6	1.2E-4
negative regulation of transcription	54	40	4.8E-6	6.9E-5
response to estrogen stimulus	21	nd	4.9E-6	-
central nervous system development	51	nd	5.2E-6	-
positive regulation of cell migration	19	nd	6.1E-6	-
negative regulation of biosynthetic process	63	47	6.3E-6	6.1E-5
negative regulation of cellular biosynthetic process	62	47	6.4E-6	3.7E-5
positive regulation of cell motion	20	nd	6.5E-6	-

negative reg. of nucleobase, nucleoside, nucleotide and nucleic acid metabolic process	58	44	6.7E-6	3.9E-5
positive regulation of cellular metabolic process	87	65	7.4E-6	5.5E-5
neurogenesis	65	nd	7.6E-6	-
positive reg. of macromolecule metabolic process	85	61	8.6E-6	2.7E-4
regulation of blood pressure	20	14	8.8E-6	4.8E-4
negative reg. of nitrogen compound metabolic process	58	44	1.0E-5	5.4E-5
regulation of phosphorus metabolic process	55	nd	1.0E-5	-
regulation of phosphate metabolic process	55	nd	1.0E-5	-
negative reg. of macromolecule biosynthetic process	60	43	1.2E-5	3.4E-4
bone morphogenesis	9	nd	1.3E-5	-
embryonic organ morphogenesis	23	16	1.8E-5	8.4E-4
myofibril assembly	9	12	1.9E-5	2.9E-10
regulation of RNA metabolic process	149	nd	2.0E-5	-
endochondral bone morphogenesis	8	nd	2.2E-5	-
vasculogenesis	12	nd	2.5E-5	-
in utero embryonic development	27	20	2.6E-5	3.2E-4
negative regulation of transcription, DNA-dependent	43	35	2.7E-5	1.9E-5
actomyosin structure organization	10	13	2.8E-5	8.6E-10
limb morphogenesis	19	nd	2.8E-5	-
cellular comp. assembly involved in morphogenesis	11	13	3.2E-5	1.8E-8
negative regulation of cellular metabolic process	72	56	3.3E-5	4.9E-5
regulation of transcription, DNA-dependent	145	nd	3.4E-5	-
negative reg. of macromolecule metabolic process	73	54	3.5E-5	2.8E-4
regulation of striated muscle tissue development	13	nd	3.6E-5	-
skeletal muscle organ development	15	nd	3.7E-5	-
negative regulation of RNA metabolic	43	35	4.0E-5	2.7E-5

process				
regulation of apoptosis	78	61	4.6E-5	4.5E-5
regulation of cell development	29	21	5.0E-5	7.8E-4
embryonic skeletal system development	16	nd	5.7E-5	-
negative regulation of signal transduction	30	nd	6.1E-5	-
regulation of programmed cell death	78	61	6.3E-5	6.1E-5
regulation of ossification	16	nd	6.6E-5	-
embryonic appendage morphogenesis	17	nd	6.7E-5	-
embryonic limb morphogenesis	17	nd	6.7E-5	-
regulation of signal transduction	82	nd	7.7E-5	-
striated muscle cell differentiation	17	20	7.8E-5	7.9E-9
generation of neurons	58	nd	8.6E-5	-
regulation of muscle contraction	15	12	8.7E-5	2.8E-4
regulation of epithelial to mesenchymal transition	6	nd	8.8E-5	-
mesoderm development	15	nd	1.0E-4	-
epithelium development	30	nd	1.1E-4	-
positive regulation of cell differentiation	30	nd	1.3E-4	-
sensory organ development	30	nd	1.5E-4	-
osteoblast differentiation	11	9	1.7E-4	4.6E-4
brain development	35	nd	1.8E-4	-
epithelial tube morphogenesis	14	nd	1.8E-4	-
muscle fiber development	10	9	1.9E-4	1.2E-4
positive regulation of cell adhesion	13	nd	2.0E-4	-
negative regulation of cell communication	31	nd	2.1E-4	-
branching involved in ureteric bud morphogenesis	8	nd	2.4E-4	-
regulation of the force of heart contraction	7	6	2.5E-4	5.0E-4
regulation of ATPase activity	7	nd	2.5E-4	-
intracellular signaling cascade	107	80	2.5E-4	7.1E-4
cell morphogenesis	40	nd	3.0E-4	-
response to nutrient	21	nd	3.4E-4	-
vasoconstriction	7	nd	3.6E-4	-
regulation of chemotaxis	9	nd	4.3E-4	-
positive regulation of response to external stimulus	13	nd	4.5E-4	-

cell morphogenesis involved in differentiation	30	nd	4.7E-4	-
endochondral ossification	6	nd	4.7E-4	-
negative regulation of cell migration	12	nd	5.9E-4	-
response to corticosteroid stimulus	15	nd	6.2E-4	-
regulation of kinase activity	39	nd	6.3E-4	-
anterior/posterior pattern formation	20	nd	7.6E-4	-
regulation of gene expression	208	nd	7.6E-4	-
positive regulation of transferase activity	29	nd	7.9E-4	-
reg. of nucleobase, nucleoside, nucleotide and nucleic acid metabolic process	205	nd	8.2E-4	-
positive regulation of phosphate metabolic process	16	nd	8.8E-4	-
positive regulation of phosphorus metabolic process	16	nd	8.9E-4	-
positive regulation of cell-substrate adhesion	8	nd	9.6E-4	-
response to progesterone stimulus	7	nd	9.6E-4	-
sarcomere organization	nd	8	-	2.2E-7
muscle thin filament assembly	nd	5	-	1.9E-5
heart septum morphogenesis	nd	5	-	1.9E-5
skeletal myofibril assembly	nd	5	-	5.4E-5
negative regulation of cell death	nd	33	-	1.5E-4
regulation of cellular ketone metabolic process	nd	11	-	1.9E-4
negative regulation of apoptosis	nd	32	-	2.4E-4
regulation of fatty acid metabolic process	nd	10	-	2.7E-4
negative regulation of programmed cell death	nd	32	-	3.1E-4
heart contraction	nd	7	-	4.0E-4
regulation of lipid metabolic process	nd	15	-	4.4E-4
cardiac cell development	nd	5	-	1.0E-4
cardiac muscle cell development	nd	5	-	1.0E-4

Cellular component

Term	Count		p-value	
	hiPS-BCs	ES-BCs	hiPS-BCs	ES-BCs
contractile fiber	33	30	4,20E-13	3,70E-14
**basement membrane**	26	16	7,40E-13	8,70E-07
contractile fiber part	31	29	2,10E-12	4,20E-14
myofibril	30	27	7,40E-12	1,40E-12
sarcomere	27	25	6,20E-11	3,70E-12
**collagen**	16	12	4,00E-10	1,70E-07
I band	17	15	7,40E-08	7,00E-08
Z disc	15	13	2,60E-07	4,60E-07
actin cytoskeleton	39	34	5,10E-07	7,60E-08
plasma membrane part	178	nd	3,40E-06	-
A band	8	6	1,90E-05	4,70E-04
**fibrillar collagen**	7	8	2,90E-05	1,90E-07
cytoplasm	490	375	1,10E-05	9,00E-07
platelet alpha granule lumen	11	10	1,10E-04	5,70E-05
cytoplasmic membrane-bounded vesicle lumen	11	10	2,10E-04	1,00E-04
sarcolemma	13	12	4,00E-04	1,10E-04
anchoring junction	24	nd	1,90E-04	-
caveola	12	nd	2,00E-04	-
cytoskeleton	112	nd	2,70E-04	-
membrane raft	21	nd	3,20E-04	-
actin filament	10	nd	5,70E-04	-
smooth muscle contractile fiber	4	nd	8,10E-04	-
actomyosin	8	nd	8,10E-04	-
cell-substrate junction	17	nd	8,80E-04	-
striated muscle thin filament	nd	6	-	3,30E-04
anchoring junction	nd	19	-	4,90E-04
cytoplasmic part	nd	251	-	4,90E-04

Molecular function

	Count		p-value	
Term	iPS-BC	ES-BC	iPS-BC	ES-BC
protein binding	605	450	1.2E-20	2.2E-17
binding	794	584	1.8E-12	4.1E-10
growth factor binding	28	19	3.7E-11	4.5E-7
sequence-specific DNA binding	78	49	6.7E-11	2.8E-5
glycosaminoglycan binding	30	16	1.9E-9	1.0E-3
ECM structural constituent	23	17	2.6E-9	6.1E-7
transcription factor activity	103	65	3.0E-9	3.4E-4
pattern binding	30	nd	1.7E-8	-
polysaccharide binding	30	nd	1.7E-8	-
cytoskeletal protein binding	62	45	4.3E-8	5.6E-6
actin binding	46	31	6.1E-8	6.7E-5
receptor binding	92	66	7.4E-8	1.4E-5
transcription regulator activity	137	94	8.6E-8	1.5E-4
SMAD binding	15	12	1.7E-7	2.4E-6
heparin binding	22	nd	4.2E-7	-
platelet-derived growth factor binding	8	7	6.6E-7	2.5E-6
transcription repressor activity	39	28	1.8E-5	5.0E-4
identical protein binding	65	53	1.9E-5	7.5E-6
structural constituent of muscle	12	12	2.3E-5	1.1E-6
calcium ion binding	84	75	3.4E-5	7.3E-8
integrin binding	13	nd	1.4E-4	-
collagen binding	10	nd	1.5E-4	-
cytokine binding	18	nd	1.9E-4	-
transforming growth factor beta receptor binding	7	6	2.9E-4	6.0E-4
protein dimerization activity	53	nd	2.9E-4	-
transcription activator activity	43	33	3.2E-4	8.7E-4
carbohydrate binding	38	nd	4.5E-4	-
structural molecule activity	57	48	7.2E-4	9.5E-5
extracellular matrix binding	8	nd	7.7E-4	-
specific RNA polymerase II transcription factor activity	nd	9	-	3.8E-4
promoter binding	nd	10	-	7.0E-4
ion binding	nd	215	-	8.4E-4
RNA pol. II transcription factor activity	nd	23	-	8.9E-4
cation binding	nd	212	-	9.1E-4

KEGG and BIOCARTA pathways

Term	Count		p-value	
	iPS-BC	ES-BC	iPS-BC	ES-BC
ECM-receptor interaction	24	18	3,40E-10	1,40E-07
Focal adhesion	36	29	4,70E-09	5,50E-08
Hypertrophic cardiomyopathy (HCM)	20	17	2,90E-07	6,60E-07
Dilated cardiomyopathy	20	18	1,10E-06	3,90E-07
Pathways in cancer	40	30	2,40E-05	3,40E-04
ALK in cardiac myocytes	13	11	3,80E-05	2,70E-05
Arrhythmogenic right ventricular cardiomyopathy (ARVC)	14	14	3,90E-04	2,20E-05
TGF-beta signaling pathway	17	nd	4,20E-05	-
MAPK signaling pathway	30	nd	1,00E-04	-
Cardiac muscle contraction	nd	13	-	7,90E-05
